# Serotonin involvement in okadaic acid-induced diarrhoea in vivo

**DOI:** 10.1007/s00204-021-03095-z

**Published:** 2021-06-20

**Authors:** M. Carmen Louzao, Celia Costas, Paula Abal, Toshiyuki Suzuki, Ryuichi Watanabe, Natalia Vilariño, Cristina Carrera, Andrea Boente-Juncal, Carmen Vale, Mercedes R. Vieytes, Luis M. Botana

**Affiliations:** 1 Departamento de Farmacología, Facultad de Veterinaria, Universidade de Santiago de Compostela, 27002 Lugo, Spain; 2 Fisheries Technology Institute, National Research and Development Agency, Japan Fisheries Research and Education Agency, Yokohama, 236-8648 Japan; 3 Departamento de Fisiología, Facultad de Veterinaria, Universidade de Santiago de Compostela, 27002 Lugo, Spain

**Keywords:** Okadaic acid, Diarrhoeic shellfish poisoning (DSP), 5-Hydroxytryptamine, Neuropeptide Y, Peptide YY

## Abstract

The consumption of contaminated shellfish with okadaic acid (OA) group of toxins leads to diarrhoeic shellfish poisoning (DSP) characterized by a set of symptoms including nausea, vomiting and diarrhoea. These phycotoxins are Ser/Thr phosphatase inhibitors, which produce hyperphosphorylation in cellular proteins. However, this inhibition does not fully explain the symptomatology reported and other targets could be relevant to the toxicity. Previous studies have indicated a feasible involvement of the nervous system. We performed a set of in vivo approaches to elucidate whether neuropeptide Y (NPY), Peptide YY (PYY) or serotonin (5-HT) was implicated in the early OA-induced diarrhoea. Fasted Swiss female mice were administered NPY, PYY(3–36) or cyproheptadine intraperitoneal prior to oral OA treatment (250 µg/kg). A non-significant delay in diarrhoea onset was observed for NPY (107 µg/kg) and PYY(3–36) (1 mg/kg) pre-treatment. On the contrary, the serotonin antagonist cyproheptadine was able to block (10 mg/kg) or delay (0.1 and 1 mg/kg) diarrhoea onset suggesting a role of 5-HT. This is the first report of the possible involvement of serotonin in OA-induced poisoning.

**Supplementary Information:**

The online version contains supplementary material available at 10.1007/s00204-021-03095-z.

## Introduction

Okadaic acid (OA) group of toxins comprise polyether fatty acids synthetized by dinoflagellates of the genera *Prorocentrum* and *Dinophysis*. Bivalves may accumulate the toxins following the consumption of this toxic phytoplankton. Therefore, OA and related compounds enter the food chain reaching humans through toxin-containing seafood ingestion causing diarrhoeic shellfish poisoning (DSP) (Yasumoto et al. 1978). DSP can be developed fast, between 30 min and a few hours afterwards. Symptomatology includes nausea, vomiting, diarrhoea and abdominal pain, achieving full recovery after 3 days (Yasumoto et al. 1978; EFSA 2008). Exposure to DSP has been frequently reported in various countries (Young et al. 2019; Vale 2020), representing the primary cause of bans on the harvesting of aquaculture in Japan and Europe (Reguera et al. 2014).

Previous studies have revealed that OA inhibits serine/threonine protein phosphatases (PPs) 1, 2A, 4, 5 and 6 activity (Bialojan and Takai 1988; Brewis et al. 1993; Chen et al. 1994; Prickett and Brautigan 2006). PPs remove a phosphate group from the phosphorylated amino acid residue of a wide variety of proteins (Yadav et al. 2017), meaning disturbance in their activity can modify downstream cellular pathways. OA in vitro has been described to induce cytoskeleton reorganization (Espina et al. 2010; Opsahl et al. 2013; Louzao et al. 2015), cell death (Dietrich et al. 2020) and cell cycle alteration (Feng et al. 2018). However, during the last decade, it has been discussed whether OA-exerted effects are fully explained by its PP inhibition (Espina et al. 2010; Munday 2013). 

Diarrhoea is defined as reduced stool consistency, increased water content and number of evacuations. A wide array of causes and pathophysiological mechanisms have been proposed for both infectious and non-infectious diarrhoea (Thiagarajah et al. 2015; Anand et al. 2016; Camilleri et al. 2017). A considerable number of those mechanisms involve neuronal activation of the Enteric Nervous System (ENS). The ENS together with parasympathetic and sympathetic innervation throughout the gastrointestinal tract coordinate and regulate essential functions regarding pancreatic secretion, gut motility, fluid secretion and nutrient absorption among others (Li et al. 2000; Hu and Spencer 2018). Within components of the ENS some members of the Neuropeptide Y (NPY) family have been closely related to functions such as fluid absorption and gastric emptying (Saria and Beubler 1985; Wang et al. 2010). These 36-aa peptides’ location include neural and endocrine components (Ekblad and Sundler 2002; Mongardi Fantaguzzi et al. 2009). For instance, NPY is expressed in different regions of the brain, but also in sympathetic neurons and in the ENS (e.g., secretomotor neurons) (Cox 2007; Mongardi Fantaguzzi et al. 2009). On the contrary, enteroendocrine L cells are the mayor contributors of Peptide YY (PYY) in the body, though it has been likewise detected in myenteric neurons and in some brain areas (Ekblad and Sundler 2002; Morimoto et al. 2008). In vitro, the DSP toxin OA downregulated NPY content and release of SH-SY5Y neuroblastoma cell line (Valdiglesias et al. 2012; Louzao et al. 2015).

Another key signalling molecule in the gut is serotonin (5-HT), a bioamine mainly expressed along the digestive tract (Erspamer and Testini 1959; Erspamer 1966; Savelieva et al. 2008; Mawe and Hoffman 2013), whose physiological functions comprise intestine fluid secretion and motility (El-Salhy et al. 2013; Mawe and Hoffman 2013; Coates et al. 2017; Camilleri et al. 2017; Hu and Spencer 2018). 5-HT is present in serotoninergic enteric neurons (Okamoto et al. 2014), though enterochromaffin cells (ECCs) are its major producers which are scattered distributed along the epithelia (Sjolund et al. 1983). ECCs act as chemosensors (Braun et al. 2007; Lund et al. 2018) and mechanosensors (Fujimiya et al. 1997; Alcaino et al. 2018), triggering a response in the underlying nerve terminals and the surrounding cells via neurotransmitters’ or hormones’ signalling (Bertrand et al. 2000; Reynaud et al. 2016; Fazio Coles et al. 2020).

Gathering the variety of pathophysiologic mechanisms resulting in diarrhoea and the important role of the ENS, we studied if OA-caused diarrhoea involves alteration of intestinal hormones (PYY) and/or neurotransmitters (5-HT and NPY). To elucidate this premise, we firstly performed an in vivo approach to determinate the dose–response of OA doses on diarrhoea outcome. Second, we assessed the effect of exogenous NPY or PYY(3–36) on OA-induced diarrhoea. Furthermore, we evaluated 5-HT implication in DSP in mice using the 5-HT_1_ and 5-HT_2_ antagonist cyproheptadine (CPH) prior to OA treatment.

## Materials and methods

### Animal model

Mouse bioassay had been an accepted method for marine biotoxins detection, though nowadays has been replaced by analytical methods on behalf of NC3R’s principles (Union 2011). Based on the Organization for Economic Cooperation and Development guidelines for acute oral toxicity studies, we decided to use female mice as an animal model (OECD/OCDE 2002). One-month-old Swiss female mice weighing between 18 and 22 g from the colonies of the University of Santiago de Compostela were employed for all the experiments described. They were kept in controlled conditions of temperature (23 ± 2 °C), humidity (60–70%) and light/dark cycles (12 h/ 12 h). Mice were placed individually on metabolic cages and fasted overnight with access to 5% glucose serum. Animals were randomly assigned to each treatment. Mice received the toxin by oral gavage at 9 a.m. (10 mL/kg body weight), moment at which food and drink were provided ad libitum. When any pre-treatment was studied, it was given by intraperitoneal injection (1% body weight) prior to the toxin. Note that the assays described hereafter were preceded by these conditions. At the end of each experiment, euthanasia by CO_2_ inhalation was conducted. All animal procedures were carried out in conformity to the European (EU directive 2010/63/EU), the Spanish legislation (Real Decreto 53/2013, Decreto 296/2008) and to the principles approved by the Institutional Animal Care Committee of the University of Santiago de Compostela under the procedure Code: 01/17/LU-002 (approved on 22 September 2017).

### Materials

Okadaic acid employed in this study was kindly provided by the National Research Institute of Fisheries Science (NRIFS) from the Fisheries Research and Education Agency (Yokohama, Japan). OA isolated from toxic dinoflagellate *Prorocentrum lima* (Suzuki et al. 2014) was quantified by PULCON method (Watanabe et al. 2016) on the quantitative NMR with an external standard. Purities (purity > 95%) were also confirmed by the NMR spectroscopy. Neuropeptide Y and Peptide YY(3–36) were purchased from TOCRIS, cyproheptadine hydrochloride sesquihydrate from Sigma-Aldrich. All chemicals employed were analytical grade from Sigma-Aldrich Quimica S.A. (Madrid, Spain).

### Dose–response of okadaic acid on diarrhoea

OA was previously reconstituted with ethanol. For administration, OA doses were prepared by serial dilutions in 0.9% saline solution. OA was given by oral gavage at 10, 50, 100, 250 and 400 µg/kg doses. Control mice received the vehicle alone. Diarrhoea onset time and diarrhoea score were registered along with the symptoms presented at 1, 3, 6, 9, 12 and 24 h. Anatomopathological examination took place when the necropsy was performed. Small and large intestines were removed and stored at − 20 °C.

### Pre-treatment in vivo studies at 6 h

NPY was previously reconstituted with milliQ water. Doses of 550 µg/kg OA and 107 µg/kg NPY were prepared by dilution of each compound in physiological solution. Four treatment groups were set: (i) control, (ii) NPY, (iii) OA and (iv) NPY plus OA. Each group was performed in duplicate. In this last case, intraperitoneal injection of NPY was performed 15 min prior to OA administration by oral gavage. The time of diarrhoea outbreak, diarrhoea score and symptomatology were registered and stools collected at 1, 3 and 6 h of treatment. At the end of the experiment, animals were subjected to necropsy. Small and large intestines were sampled and kept at − 20 °C.

A similar approach was performed for PYY(3–36) pre-treatment studies. PYY(3–36) was also reconstituted with milliQ water. The peptide was diluted in physiological solution to reach 1 mg/kg PYY(3–36). Mice were split into four groups comprising control, PYY(3–36), OA and PYY(3–36) plus OA. In this last group, PYY(3–36) was given by intraperitoneal injection 15 min before oral administration of 550 µg/kg OA. Same performance and data as in the previous experiment were obtained, ending the experiment at 6 h post-toxin administration.

CPH pre-treatment approach was in line with the previous ones. CPH was first reconstituted with ethanol. CPH was diluted in physiological solution to prepare the dose 3 mg/kg. Different sets of animals were given vehicles, CPH or OA each alone or CPH plus OA. Mice were first injected CPH intraperitoneally 30 min prior to 250 µg/kg OA by oral gavage. Same data as in the previous approaches were also collected.

### NPY or PYY(3–36) pre-treatment of mice in 2 h experiments

Preparation of treatments and administration were performed as described in the preceding assessment. The peptides were each diluted in physiological solution to obtain 107 µg/kg NPY and 1 mg/kg PYY(3–36). NPY or PYY(3–36) was given intraperitoneal 15 min before oral administration of 250 µg/kg OA. During the following 2 h, the same data as detailed in the above experiment were obtained.

### Dose–response of cyproheptadine in vivo at 2 h

Doses of 0.1, 1, 3 and 10 mg/kg CPH were tested as a pre-treatment to 250 µg/kg OA for 2 h. The experimental development was as detailed above. Dosages were diluted in physiological solution. OA was given by oral gavage 30 min after CPH dose was injected via intraperitoneal. Mice were observed, stools were collected, diarrhoea score and time of onset were measured 2 h of treatment. Then necropsy was performed and the gut was removed and stored at −20 °C.

### Short-time exposure CPH dose–response

CPH and OA doses were prepared as described in previous sections. In this case, animals were split in six groups: (i) control, (ii) CPH, (iii) OA, (iv) 0.1 mg/kg CPH plus OA, (v) 1 mg/kg CPH plus OA and (vi) 6 mg/kg CPH plus OA. CPH was given via intraperitoneal 30 min before administering 250 µg/kg OA by oral gavage. Mice receiving the toxin alone were first treated, the time of diarrhoea onset was set as the end of the experiment for the remaining treatments. Same performance and data as in the previous experiment were obtained, but for 30 min post-toxin administration.

### Diarrhoea score

To assess the differences in terms of how severe the diarrhoea was, we designed a scoring system (Table 1), meaning 0 normal faeces; 1 soft faeces; 2 shapeless soft faeces; 3 watery diarrhoea; 4 having for more than once diarrhoea. To be considered a different time of diarrhoea, it was required to be at least 20 min past the last defecation.

**Table 1:** Diarrhoea scoring system criteria

0	Normal faeces
1	Soft faeces
2	Shapeless soft faeces
3	Watery diarrhoea
4	Watery diarrhoea repeatedly (taking into account 20 min between each time)

^a^A score equal or higher than 2 is considered diarrhoea

### Neuromodulators’ detection

Small intestine (ileum) and large intestine (proximal colon) were first extracted. Samples were cleansed in ice-cold PBS and weighted immediately afterwards. PBS was added (1:9 w/v) and tissues were homogenized and sonicated. Finally, they were centrifuged for 5 min at 10,000×*g* at 4 °C and stored at − 20 °C. When required, extracts were diluted for the compounds’ concentration to fall within the linear range of the standard solutions. In all cases, absorbance was measured in a Multi-mode Microplate Reader Synergy 4 (Biotek).

NPY was measured in samples from OA dose–response at 24 h and from 6 h NPY pre-treatment studies. Enzyme-linked Immunosorbent Assay (ELISA) Kit for Neuropeptide Y from Cloud Clone Corp. was employed. The range of detection was 2.47–200 pg/mL and absorbance was measured at 450 nm.

PYY was analysed in mice intestines from OA dose–response at 24 h and from 6 h PYY(3–36) pre-treatment experiments. ELISA Kit for Peptide YY (Cloud-Clone Corp.) was used. The detection range was 12.35–1000 pg/mL and absorbance was read at 450 nm.

5-HT was determined in samples stored from CPH pre-treatment (6 h), dose–response of CPH (2 h) and short time exposure CPH dose–response experiments. The Serotonin ELISA kit from Enzo Life Sciences was used for 5-HT detection. The range of detection was 0.49–500 ng/mL and absorbance was read at 405 nm.

### Statistical analysis

Graphpad Prism and RStudio were employed to perform the statistical analyses. First, the distribution and homoscedasticity of the data set were tested. If it followed a normal distribution, *t* test was performed to compare two treatments or one-way ANOVA plus Bonferroni multiple comparison test in the case more groups were analysed. Conversely, under no normal distribution of data, Mann–Whitney test or Kruskal–Wallis test followed by Bonferroni multiple comparison test was conducted. The significance threshold was set at *P* < 0.05.

## Results

### Dose–response of okadaic acid

To determine at which concentration OA is able to trigger diarrhoea, several doses of the toxin (10, 50, 100, 250 and 400 µg/kg) were administered to mice that were observed for 24 h. During the experiment, the symptomatology was monitored in detail at 1, 3, 6, 9, 12 and 24 h of OA treatment. No symptoms were detected in 10 µg/kg treated mice throughout the experiment. In the case of 50 µg/kg dose, these animals did not present any symptoms, but one mouse alone had soft faeces at 12 h, being normal at 24 h. Treatments of 100, 250 and 400 µg/kg induced squint-eyes, piloerection, spasms, cyanosis and even death for the highest dose (Table 2). This approach allowed to register the time at which mice recovered, i.e., had no symptoms. Clinical signs were noted at 1 h for 100 µg/kg OA treatment, being absent at 3 h. None of the reported symptoms were identified at 9 h for 250 µg/kg OA. Finally, no mice that received 400 µg/kg OA reached the 24 h of treatment.

**Table 2:** Symptomatology of 24 h OA-treated animals (%)

Symptoms	Control	OA (µg/kg)				
		10	50	100	250	400
Apathy	0	0	0	33.3	100	50
Piloerection	0	0	0	33.3	66.7	50
Cyanosis	0	0	0	0	0	50
Spasms	0	0	0	33.3	66.7	50
On hind legs	0	0	0	66.7	33.3	0
Squint-eyes	0	0	0	66.7	66.7	50
Diarrhoea	0	0	0	100	100	100
Mortality	0	0	0	0	0	100

**Fig. 1:**
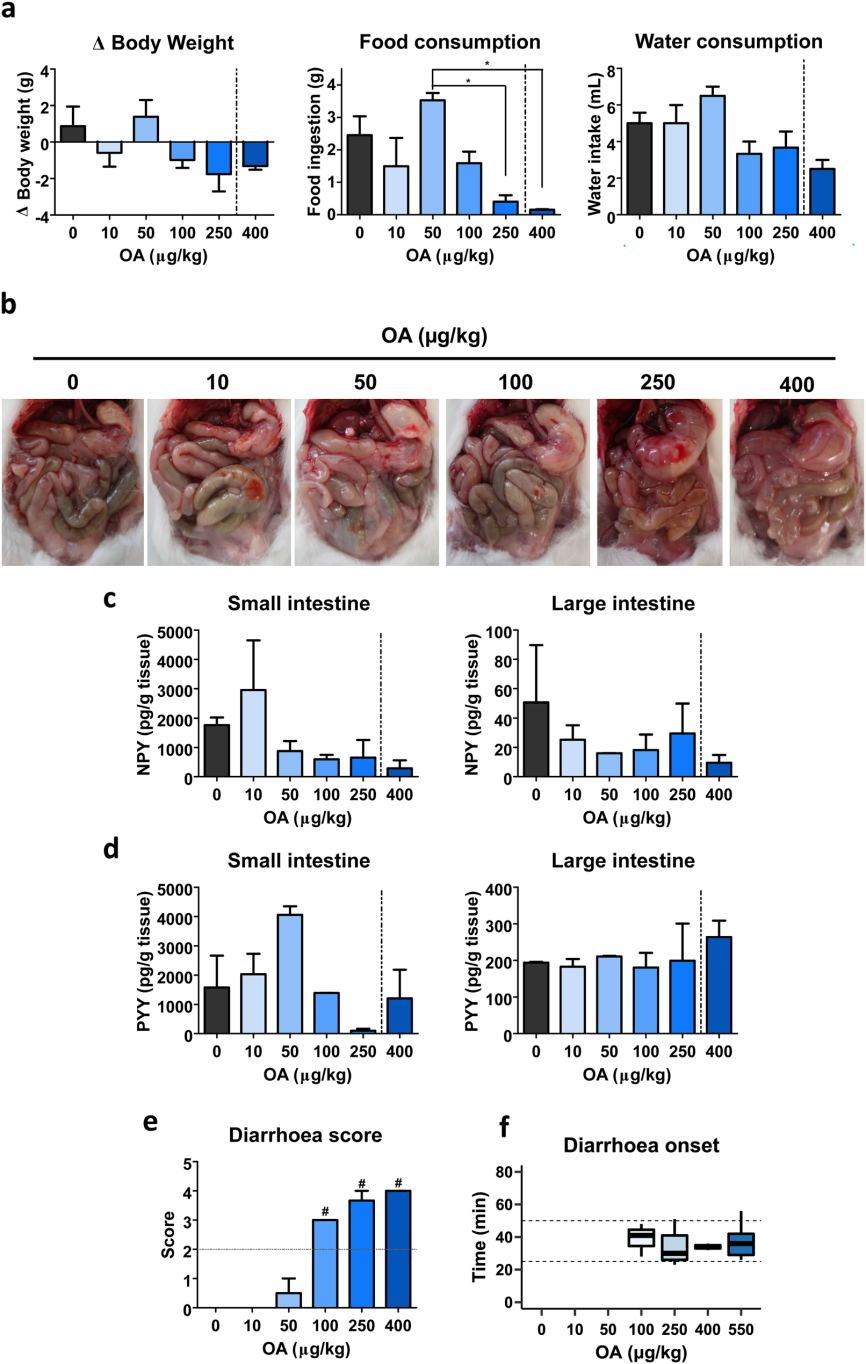
Dose–response for diarrhoea induced by doses from 10 to 400 µg/kg okadaic acid in a 24 h study period. **a** Variation on mice body weight, food and water intake. **b** Representative images of the mice abdominal cavity at the end of the experiment. Image corresponding to 400 µg/kg OA was taken 11:45 h after toxin administration, time at which the animal died. **c** Neuropeptide Y measured in small and large intestines. **d** Peptide YY detected in small and large intestines. **e** Diarrhoea score. **f** Diarrhoea onset time. Inner box line indicates median and dashed lines are set at 25 and 50 min. Graphs **a**, **c**, **d** and **e** display mean ± SEM (*n* = 3). Since no animal treated with 400 µg/kg accomplished the experimental time, data for this dose are separated by dotted line. Statistical analysis was conducted with one-way ANOVA–Bonferroni multiple comparison test. In **e**, treatments with ‘#’ over the bar are significantly different (*P* < 0.001) from those with a ‘ + ’. Significance is indicated with asterisks over the line between treatments, so that *P < 0.05, otherwise non-significant

Variations in body weight after 24 h of OA treatment were measured, as well as food and water consumption during the experiment (Fig. 1a). Both body weight variation and food intake display a similar pattern.

At the end of the experiment, mice were subjected to necroscopic analysis, focusing on anatomopathological evaluation of the gastrointestinal tract (Fig. 1b). While 10, 50 and 100 µg/kg OA examination revealed no differences with control, 250 µg/kg dose was featured by a swollen stomach and fluid accumulation in the small intestine. In the case of 400 µg/kg OA-treated mice, swollen stomach accompanied by moderate to strong fluid accumulation in the small intestine were observed for all animals.

Neuropeptides like NPY and PYY are involved in the regulation of nutrients absorption and exert a protective role (Moriya et al. 2010; Tough et al. 2011). Thus, we aimed to elucidate how OA affects NPY and PYY along the gut in vivo after 24 h treatments. OA reduces NPY in the small intestine and in large intestine (Fig. 1c). Only 250 µg/kg OA treatment decreases PYY in small intestine while large intestine’s PYY is not affected by OA at any of the given doses at 24 h (Fig. 1d).

The evaluation of clinical signs was focused on diarrhoea (percent of mice with this symptom, diarrhoea onset time and diarrhoea score). To assess the severity of diarrhoea, faeces from each mouse were scored as described above (Table 1). Based on diarrhoea score criteria, there is a dose-dependent increase up to the maximal punctuation (Fig. 1e). Control, 10 µg/kg and 50 µg/kg OA had normal faeces, except for one mouse that received the latter dose. Then the lowest dose tested for developing diarrhoea was 100 µg/kg, with several defecations. Since neither control, 10 nor 50 µg/kg treated mice had diarrhoea, no onset is represented regarding these treatments (Fig. 1f).

To further assure diarrhoea in the following approaches, we also included the dose: 550 µg/kg OA (Fig. 1f). We found no significant differences in diarrhoea onset between any of the OA doses that triggers the symptom, showing an all-or-none response.

### NPY effect on OA poisoning in 6 h experiments

Based on how OA affected in vitro NPY expression in addition to the pro-absorptive role of the neuropeptide, we designed a 6 h experiment in which mice were intraperitoneally administered NPY prior to OA treatment. Due to the lack of differences between doses in diarrhoea onset (Fig. 1f) along with the evaluation of a dose closer to the previously described oral LD_50_ for OA (760 µg/kg) (Abal et al. 2018), we considered 550 µg/kg to be suitable to perform this assessment. The time of the experiment, 6 h, was selected to assure not just the inhibition of diarrhoea detection, but any delay in diarrhoea onset. Symptomatology of the animals was recorded during the experiment (Table S1). Most animals exhibited a variety of symptoms such as piloerection and squint-eyes. Symptoms were still observed in mice treated with OA alone or NPY-OA at the end of the experiment. It should be remarked the fact that all mice treated with the toxin or with the combination of both, OA and NPY (NPY-OA), developed diarrhoea.

Body weight variations (Fig. S1a), food (Fig. S1b) and water intake (Fig. S1c) were measured. Necropsy of all animals revealed that the toxin induced fluid accumulation along the small intestine, being modestly improved by NPY (Fig. S1d).

**Fig. 2:**
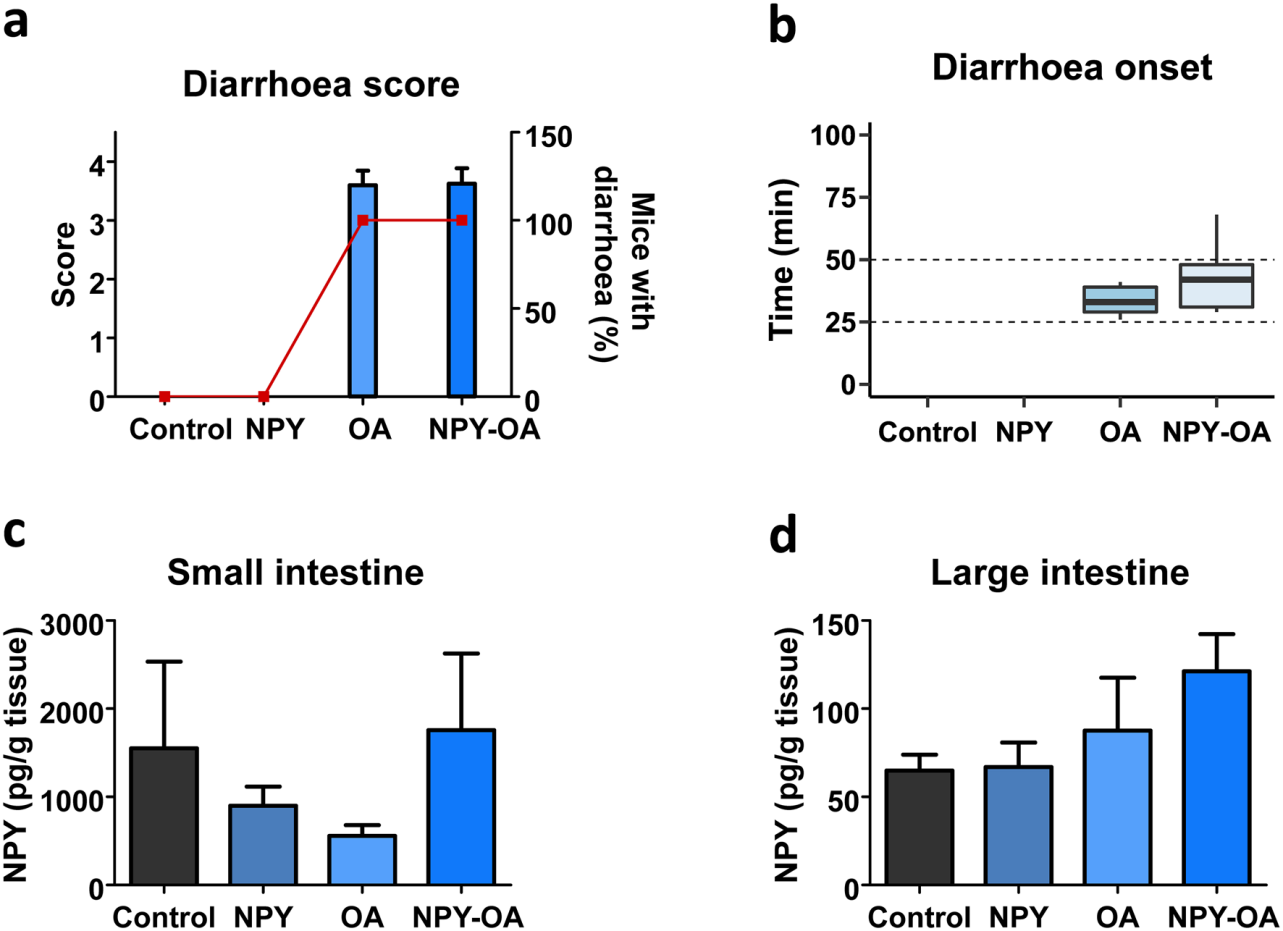
NPY effect on OA-induced diarrhoea and changes in NPY in the gut at 6 h experiment. Animals were given 107 µg/kg NPY 15 min prior to 550 µg/kg OA administration. **a** Diarrhoea score (bars) and percent of mice that developed diarrhoea (closed squares). **b** Time of diarrhoea outbreak, values are expressed as boxplot showing the median. **c**–**d** NPY concentration in small (**c**) and large intestines (**d**). Data are mean ± SEM (*n* = 4 performed in duplicate). Student t test resulted in no significant differences (**b**). For **c** and **d**, statistical analyses were performed by one-way ANOVA and Bonferroni multiple comparison test with no significant differences

The type of diarrhoea was evaluated with the diarrhoea score (Fig. 2a). Pre-treatment with NPY did not modify OA-induced diarrhoea nor supressed it (Fig. 2a). Diarrhoea onset time displays a short, non-significant, delay with NPY pre-treatment (OA 33 ± 3.4 min; NPY-OA 43 ± 4.9 min) (Fig. 2b).

NPY was measured in the intestine of these animals 6 h after toxin administration (Fig. 2c, d). OA reduces NPY concentration in the small intestine; meanwhile, in NPY-OA-treated animals’ NPY resembles control concentration (Fig. 2c). Conversely, the same treatment seems to induce a modest rise of NPY in the large bowel (Fig. 2d).

### PYY(3–36) effect on OA poisoning in 6 h experiments

The enteric nervous system plays a vital role in the response to various gastrointestinal stimuli. The peptides of this nervous system regulate gastrointestinal movement, secretion, absorption and other complex functions through endocrine, paracrine and neuronal actions. Both NPY and Peptide YY are important enteric peptides. The observation of a soft effect of NPY over OA intoxication led us to study PYY. Y_2_ receptors are not only in nerve terminals around myenteric neurons, but also in mucosa and muscle layers and its agonist PYY(3–36) has additionally been related to a clear anti-diarrhetic effect (Moriya et al. 2010; Tough et al. 2011). Thus, we aimed to check whether this agonist had the ability to relieve OA-induced diarrhoea. PYY(3–36) (1 mg/kg) was administered prior to OA (550 µg/kg) was given*.* Symptomatology was monitored along 6 h (Table S2). Apathy, piloerection and squint-eyes were developed following toxin treatment, alone or in combination with PYY(3–36) [PYY(3–36)-OA]. Both OA and PYY(3–36)-OA-treated mice still presented symptoms at the end of the experiment.

Subsequently, the balance of body weight (Fig. S2a), food (Fig. S2b) and water consumption (Fig. S2c) were measured. Anatomopathological evaluation revealed swollen stomachs and small intestine fluid accumulation of the toxin-treated animals, with or without PYY(3–36) (Fig. S2d).

**Fig. 3:**
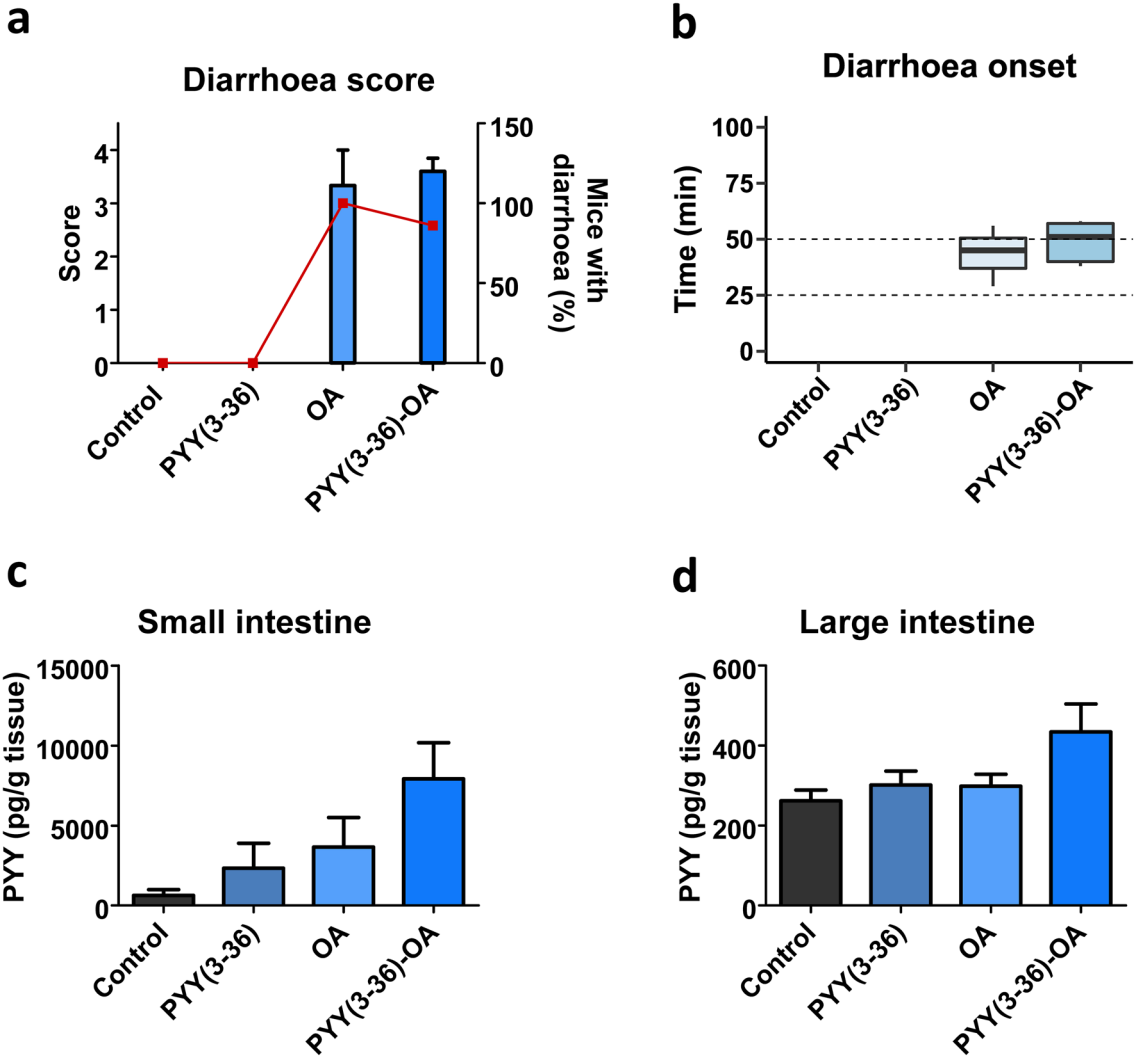
PYY(3–36) effect on OA-induced diarrhoea and changes in PYY in the gut at 6 h. PYY(3–36) (1 mg/kg) was given to mice 15 min previous to OA (550 µg/kg). **a** Diarrhoea score (bars) along with percent of mice with diarrhoea (closed squares). **b** Time of diarrhoea onset; median is shown within each box. **c**–**d** PYY detected in small (**c**) and large (**d**) intestine of mice 6 h after treatments. Data are presented as mean ± SEM (*n* = 3 performed in duplicate). Student *t* test (**b**) or one-way ANOVA (**a**, **c** and **d**) were performed with no statistical significance detected in either case

Diarrhoea score shows no differences between OA and PYY(3–36)-OA (Fig. 3a). Diarrhoea outbreak of PYY(3–36)-OA-treated mice displays a slight time delay when compared to OA alone [OA 43 ± 7.8 min; PYY(3–36)-OA 49 ± 4.2 min] (Fig. 3b). Still, it is a remarkable fact that 100% of OA-treated mice presented diarrhoea; meanwhile, 85.7% of PYY(3–36)-OA-treated mice showed this symptom (Table S2 and Fig. 3a).

It was then of interest to analyse the effect of the toxin on PYY concentration (Fig. 3c, d). The amount of PYY measured in small intestine has a remarkable non-significant increase in PYY(3–36)-OA-treated mice (Fig. 3c). In the large intestine, PYY(3–36)-OA treatment induces a minor rise compared to control (Fig. 3d).

### NPY and PYY(3–36) pre-treatment effect on OA poisoning in 2 h experiments

Neither NPY nor PYY(3–36) modified OA poisoning, still it was of interest to assure that this was independent of OA dose. Subsequently, 250 µg/kg OA was chosen to both assure diarrhoea (Fig. 1e–g) and avoid mortality (Table 2). Since diarrhoea outbreak appears in less than 2 h, this was set as the endpoint of the experiment. During this time, monitoring of clinical signs identified symptoms such as piloerection or squint-eyes in all treatments involving the toxin (Table S3).

Body weight variation (Fig. S3a) and food intake (Fig. S3b) were measured showing no significant differences between treatments. At the end of the experiment, macroscopic evaluation of the abdominal cavity was performed (Fig. S3c). Animals treated with OA, NPY-OA and PYY(3–36)-OA revealed fluid accumulation mainly along the intestine even some mice that had no stools. Large intestines were also removed and examined separately (Fig. S3c). Diarrhoeic content was observed in OA-treated mice alone or in combination with NPY or PYY(3–36).

**Fig. 4:**
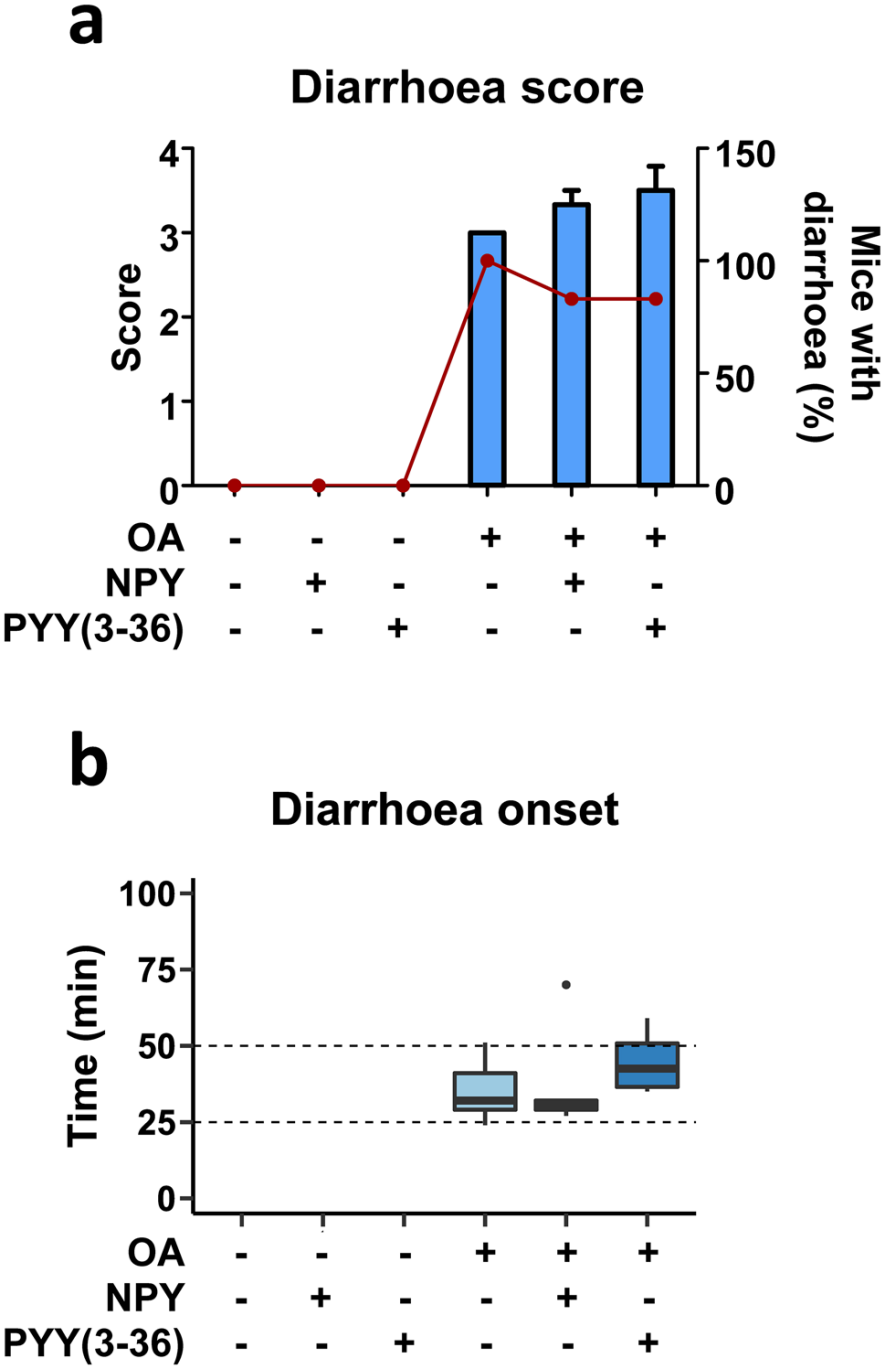
NPY or PYY(3–36) pre-treatment effects on OA diarrhoea during 2 h experiments. NPY (107 µg/kg) or PYY(3–36) (1 mg/kg) were administered 15 min prior to receiving OA (250 µg/kg). **a** Diarrhoea score (bars) and percent of mice that developed diarrhoea (closed circles). **b** Diarrhoea onset time, boxes show data distribution, indicating the median as the line within each box. Data are expressed as mean ± SEM (*n* = 3 with duplicates). One-way ANOVA was conducted resulting in no significant differences in either case

Diarrhoea was also evaluated (Fig. 4). The type of diarrhoea developed by animals pre-treated with either NPY or PYY(3–36) was not different from that induced by OA alone (Fig. 4a). Note that animals treated with control, NPY or PYY(3–36) had no stools during the experimental time, so the score is 0 (Fig. 4a) and no diarrhoea onset is shown for these treatments (Fig. 4b). Regarding onset time of diarrhoea, only a slight non-significant delay is observed in PYY(3–36) pre-treatment [OA 35 ± 2.1 min; NPY-OA 38 ± 8.1 min; PYY(3–36)-OA 45 ± 5.5 min] (Fig. 4b).

### CPH effect on OA poisoning

**Fig. 5:**
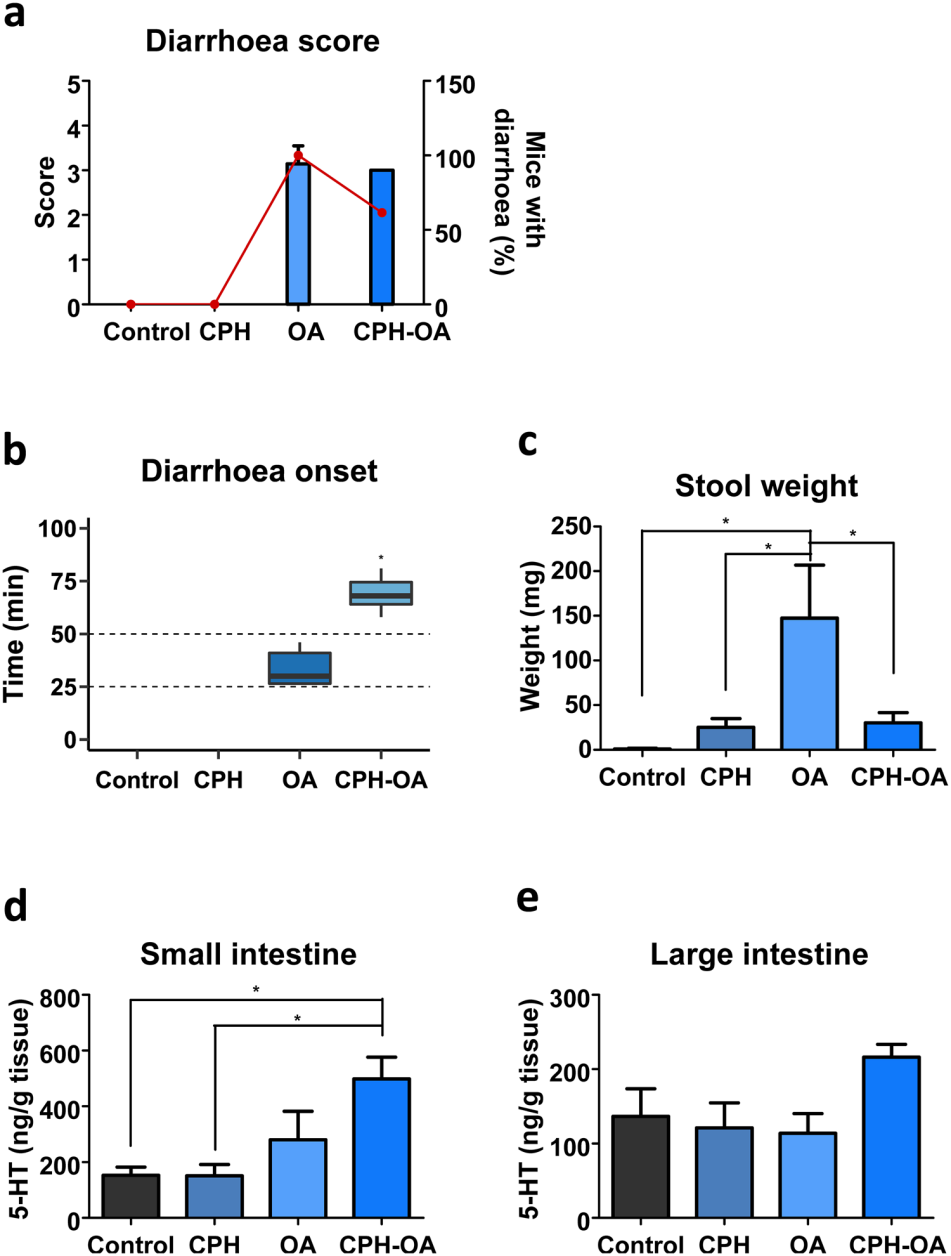
CPH pre-treatment effect on OA-induced diarrhoea (6 h). Mice were treated with CPH (3 mg/kg) 30 min before OA (250 µg/kg) administration. **a** Diarrhoea score (bars) along with the percent of animals developing diarrhoea (closed circles). **b** Time of diarrhoea outbreak. Inside boxes median is indicated. **c** Faeces wet weight. **d**–**e** 5-HT concentration measured in small (**d**) and large intestines (**e**) of mice. Mean ± SEM (*n* = 7 of duplicates) are presented. One-way ANOVA (**a**, **c**) or Kruskal–Wallis (**d**–**e**) followed by Bonferroni multiple comparison test was performed. Significance is indicated by asterisks over the line between groups, such as *P < 0.05. Student *t* test comparing OA with CPH-OA was conducted to study diarrhoea onset (**b**), resulting in *P < 0.05

The lack of a robust reaction to Y receptor ligands led us to study secretory pathways instead of pro-absorptive mechanisms. Serotonin is a key signalling molecule that mediates physiological processes in the gut and its release is stimulated by diarrhoeagenic compounds. Cyproheptadine (CPH), an inverse agonist/antagonist of 5-HT receptors 1 and 2, has been described to elicit a response at the level of other antisecretory drugs (Meddah et al. 2014). Consequently, to assess the role of 5-HT on OA mechanism to induce diarrhoea, we administered 3 mg/kg CPH before OA treatment. The set of symptoms developed were observed for 6 h and were similar in mice that received OA and OA with CPH (CPH-OA) (Table S4). Diarrhoea was the representative symptom and it should be highlighted that CPH pre-treatment reduced the prevalence from 100% of the toxin alone to 61.5% (Table S4 and Fig. 5a).

As in previous approaches, body weight variations (Fig. S4a) as well as food (Fig. S4b) and water intake (Fig. S4c) were measured. Food intake was reduced in groups of mice that received the toxin or CPH-OA (Fig. S4b). Macroscopic evaluation of the abdominal cavity revealed mild fluid accumulation in the small intestine of animals that received OA, resembling that of CPH-OA treated mice (Fig. S4d). Large intestines were removed and examined (Fig. S4d). OA large intestines were featured by diarrhetic content; meanwhile, CPH pre-treatment helped nearly restore normal intestinal content.

With regard to diarrhoea measured parameters, no significant difference in diarrhoea score was detected between OA and CPH-OA treated mice (Fig. 5a). Conversely, onset of diarrhoea is significantly delayed by CPH administration [OA 34 ± 3.7 min; CPH-OA 69.1 ± 3.2 min] (Fig. 5b). OA induced a stark increase in faeces wet weight that decreased significantly with CPH pre-treatment (Fig. 5c).

Intestine’s 5-HT was quantified in mice samples (Fig. 5d, e). OA and CPH-OA induced 5-HT increase in the small intestine (Fig. 5d). Although in large intestine, CPH-OA treatment shows a non-significant increase over the other treatments (Fig. 5e).

### Dose–response of CPH in OA poisoning

The suppression of OA-triggered diarrhoea led us to perform a dose–response study. Since CPH effect occurs within 2 h after toxin administration, experimental time was reduced to 2 h. Here we assessed 0.1, 1, 3 and 10 mg/kg CPH as a pre-treatment for 250 µg/kg OA. Clinical signs developed by each group of treatment were monitored (Table S5). It is noteworthy that diarrhoea was present in all mice administered with OA, but was absent in those pre-treated with 3 or 10 mg/kg CPH.

Body weight variation (Fig. S5a) as well as food ingestion (Fig. S5b) were measured. It was observed a tendency in weight lost and reduced food intake. On the contrary, anatomopathological examination provides information regarding the gastrointestinal tract at macroscopic level (Fig. S5c). Fluid accumulation in the stomach and intestine was observed for OA-treated mice. In animals pre-treated with 0.1, 1 and 3 mg/kg CPH, OA still induced fluid accumulation in the intestine and stomach. However, mice with 10 mg/kg CPH pre-treatment displayed an ameliorated fluid content in the intestine and a degree of solid content in the stomach. Complementary, large intestine state was evaluated (Fig. S5c), diarrhetic content is appreciated in mice administered the toxin alone or in combination with 0.1 mg/kg CPH. A mild improvement can be appreciated in the intestine of mice pre-treated with 1 mg/kg CPH, being back to normal with 3 and 10 mg/kg CPH.

**Fig. 6:**
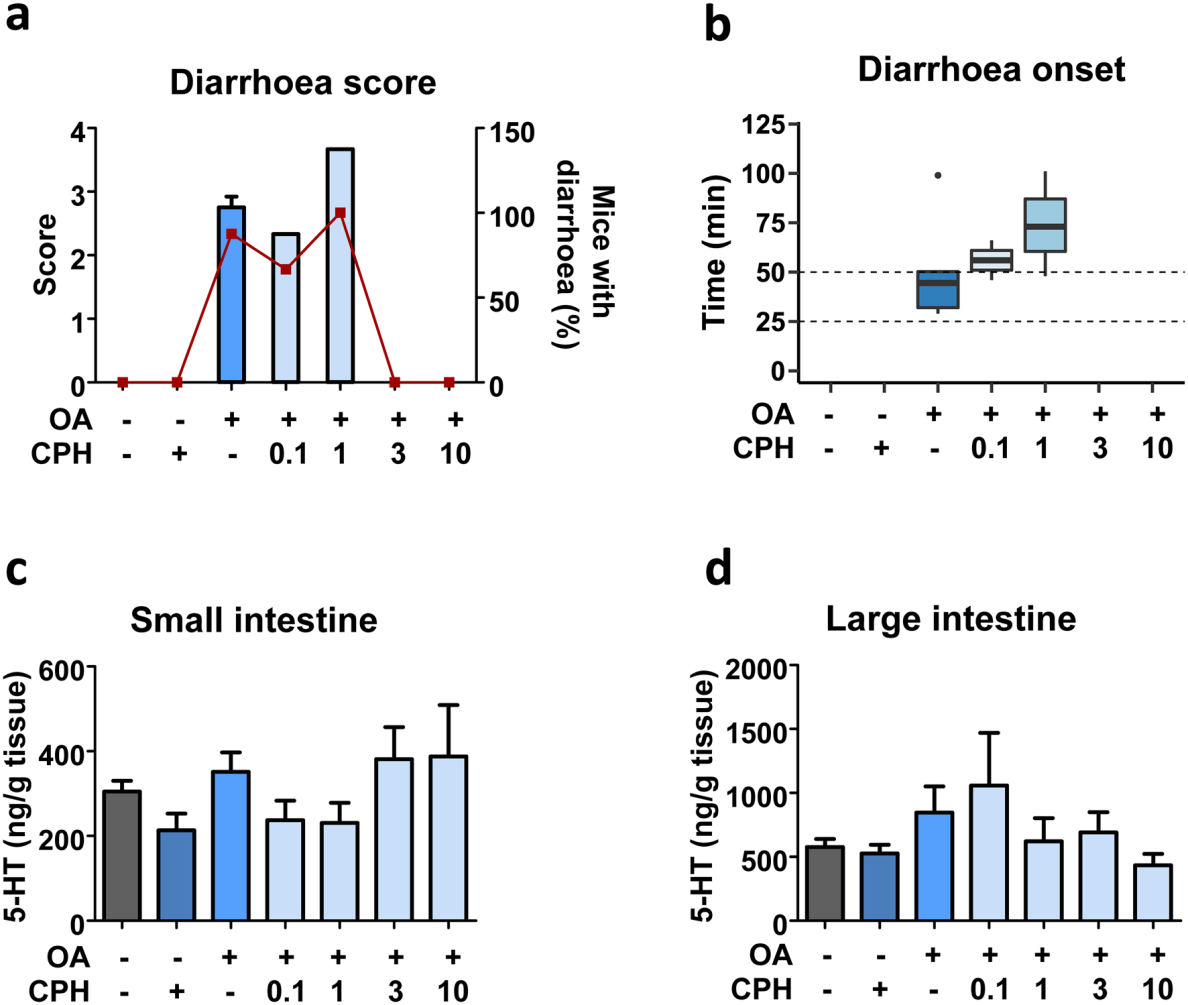
Dose-dependent effect of CPH on OA-induced diarrhoea (2 h). Mice received 0.1, 1, 3 or 10 mg/kg CPH 30 min before the administration of 250 µg/kg OA. **a** Diarrhoea score (bars) and percent of mice developing diarrhoea (closed squares). **b** Time of diarrhoea onset; line within each box represents the median. **c**–**d** 5-HT concentration measured in small (**c**) and large (**d**) intestines. Data are shown as mean ± SEM (n = 3 of duplicates). One-way ANOVA (**a**–**b**) or Kruskal–Wallis (**c**–**d**) and Bonferroni multiple comparison tests were performed as statistical analyses. No significant differences were detected

In a further evaluation of diarrhoea, score and onset were studied (Fig. 6a, b). Diarrhoea score varies between 2.7 and 4 (Fig. 6a). There was a delay in diarrhoea onset when mice were pre-treated with 0.1 or 1 mg/kg CPH (Fig. 6b). In opposite, 3 and 10 mg/kg CPH pre-treatment blocked OA-induced diarrhoea (Fig. 6a). It should be taken into consideration the fact that control mice, or mice treated with CPH or OA plus 3 or 10 mg/kg CPH had no stools along the experiment (Fig. 6b).

We examined if different doses of CPH had any effect on 5-HT concentration (Fig. 6c, d). Overall, no significant variations were observed in small intestine’s 5-HT (Fig. 6c). In the large intestine, there was a dose-dependent no significant decrease in 5-HT (Fig. 6d).

### Evaluation of CPH doses effect when OA induced diarrhoea

**Fig. 7:**
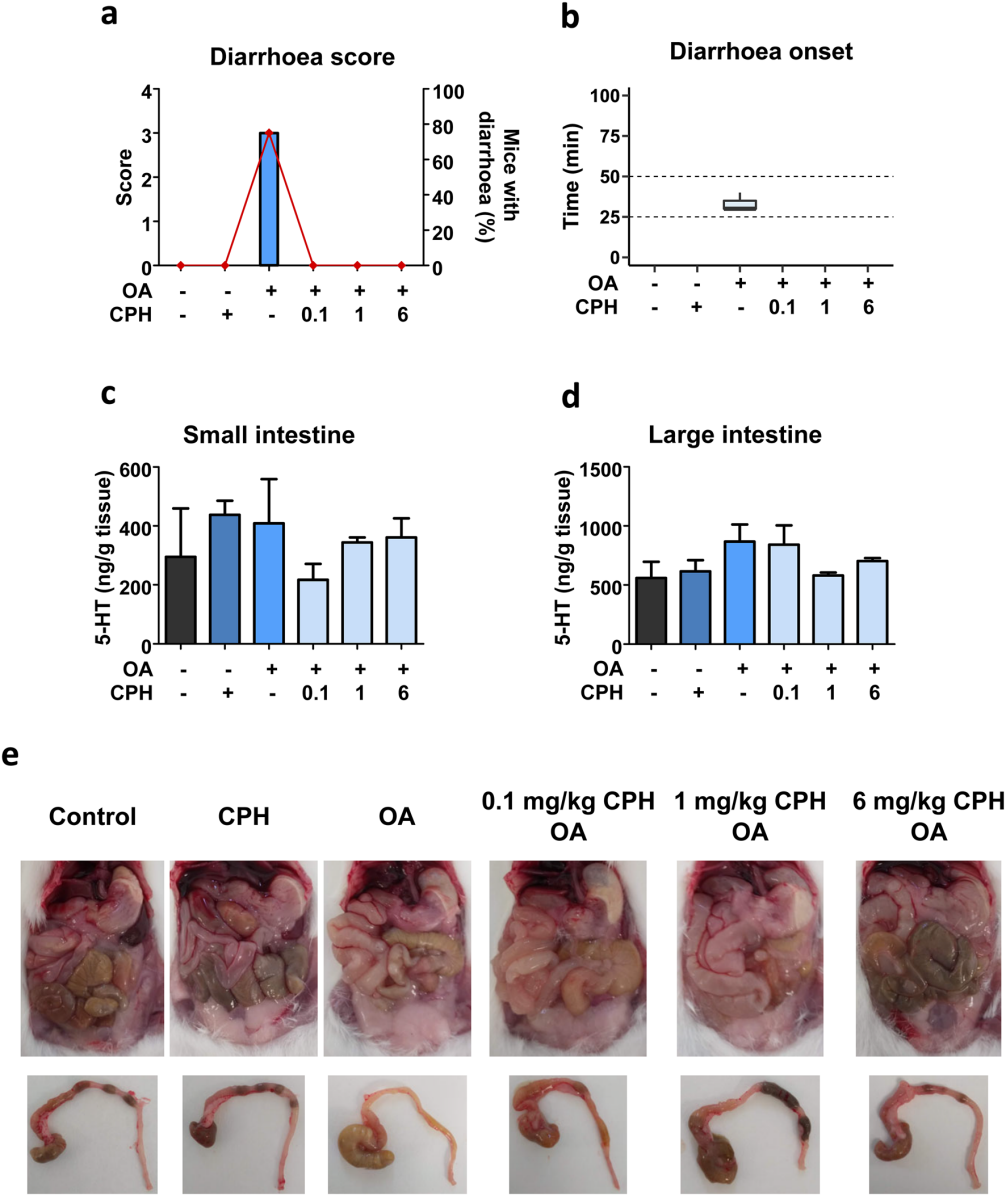
CPH pre-treatment effect on OA-induced diarrhoea onset. Mice were pre-treated with 0.1, 1 or 6 mg/kg CPH and 30 min later treated with 250 µg/kg OA. The end of the experiment was set at the time OA triggered diarrhoea. **a** Diarrhoea score (bars) and percent of mice having diarrhoea (closed diamonds). All animals had normal or no stools (score of 0), but for those treated with OA. **b** Time of diarrhoea onset presented as a boxplot indicating the median inside the box. **c**–**d** 5-HT concentration detected on small (**c**) and large (**d**) intestines of mice. **e** Representative images of abdominal cavity and large intestine. Mean ± SEM (*n* = 3 of duplicates) is presented. Kruskal–Wallis (**c**) or one-way ANOVA (**d**) resulted in no significant differences

Based on the previous results, it was interesting to assess the effect of CPH when diarrhoea was triggered by OA. To elucidate this, first animals were pre-treated with different doses of CPH and 30 min later treated with 250 µg/kg OA and euthanised at the time when diarrhoea should appear. Average of OA-triggered diarrhoea outbreak was 33 ± 2.3 min (Fig. 7b); therefore, this time was set as the end of the experiment. Notice the fact that only OA-treated mice had diarrhoea as shown by diarrhoea score (Fig. 7a).

During the brief experimental time, symptoms developed were observed (Table S6). No mice treated with any dose of CPH plus OA developed diarrhoea.

Both mice body weight balance (Fig. S6a) and food consumption (Fig. S6b) display subtle variations due to the shortened experimental time.

Determination of 5-HT in the small (Fig. 7c) and large intestine (Fig. 7d) was then conducted. OA increased 5-HT in the small intestine (non-significant) that decreased with CPH pre-treatments (Fig. 7c). Conversely, the toxin induced a modest 5-HT rise not observed in the presence of 1 nor 6 mg/kg CPH in the large intestine (Fig. 7d).

Necroscopy of mice allowed the evaluation of the OA-induced effects along the gastrointestinal tract (Fig. 7e). No clear differences between administration of OA alone or in combination with 0.1 or 1 mg/kg CPH were detected. Yet, pre-treatment with 6 mg/kg CPH did improve the gastrointestinal tract aspect bringing it closer to that of control. In the large intestine, diarrhetic content was noticed in mice treated with OA and OA plus 0.1 mg/kg CPH, which was partially reversed by 1 mg/kg CPH (Fig. 7e). As described for the small intestine, 6 mg/kg CPH large intestines resembled those of control.

## Discussion

Microalgae of the genera *Dinophysis* and *Prorocentrum* produce OA and form hazardous blooms leading to adverse environmental consequences associated with the declines of zooplankton populations (Gong et al. 2021). Besides, the consumption of seafood contaminated by OA or their structural derivatives, dinophysis toxins, causes DSP (Yasumoto et al. 1978). Due to the human health concerns associated with DSP, OA group of phycotoxins are tightly regulated by European Union legislation (Union 2011). Even though many in vitro and in vivo studies have been performed with OA, there are still many gaps about the targets involved in its acute oral toxicity (Louzao et al. 2021; Huguet et al. 2020; Dietrich et al. 2019; Reale et al. 2019; Tripuraneni et al. 1997; Ferron et al. 2014; Vilarino et al. 2018). It is stated in the literature that okadaic acid group of toxins are inhibitors of Ser/Thr protein phosphatases 1 (PP1) and 2A (PP2A) which play many roles in the cell (Takai et al. 1992). However, some challenging reports arise the possibility of different action mechanisms triggering gastrointestinal symptoms (Vilarino et al. 2008; Espina et al. 2010; Munday 2013). However, taking into account the rapid onset of this main symptom, the involvement of the enteric nervous system should not be ruled out. Thus, it is of great interest to elucidate the specific signalling pathway resulting in OA-induced diarrhoea.

We designed an OA dose–response study to characterize, among others, effects caused by the toxin for 24 h and particularly diarrhoea onset. In some mice, OA induced various symptoms such as on hind legs, squint-eyes, apathy, piloerection or spasms, but all animals that showed clinical signs of intoxication developed diarrhoea. The toxic effects of OA included fluid accumulation in the gastrointestinal tract, and even death at high doses. To perform a risk assessment, parameters as No-Observed-Adverse-Effect-Level (NOAEL) derived from the estimated exposures have been used to define Acute reference dose (ARfD) for humans. In our hands, 50 µg/kg OA was the highest administered dose at which no symptom or clinical sign was observed. This NOAEL agrees with the one proposed by EFSA in mice (2008). Besides our study revealed that 100 µg/kg OA was the lowest dose developing symptoms according to the LOAEL (Lowest-Observed-Adverse-Effect-Level) previously indicated in humans (Toyofuku 2006). Administration of doses equal or higher than 100 µg/kg OA triggers diarrhoea reaching the score of 3 or 4, with no differences in time onset indicating an all-or-none response. This could suggest a neuronal pathway in OA pathophysiology as was previously published in relation to the intestine water absorption-secretion balance (Delbro and Lange 1997). Recently, OA has also been involved in oxidative stress and inflammation pathways activation in enteric glial cell culture (Reale et al. 2019).

Diarrhoea represents an increase in water content of the stool and in the frequency of evacuation and mainly results from dysregulation of either intestinal secretory function or colonic motor function (Moriya et al. 2010). These intestinal activities are regulated by the enteric nervous system and implicate the Neuropeptide Y family as mediators (Vona-Davis and McFadden 2007). This family includes Neuropeptide Y (NPY) and Peptide YY (PYY) that act as hormone and/or neurotransmitters/neuromodulators. They exert their functions through binding to Y-receptor subtypes of transmembrane-domain G-protein-coupled receptors (El-Salhy et al. 2020). PYY and NPY have similar biological effects and bind to and activate receptors Y_1_ and Y_2_ localized in epithelial cells and submucosal and myenteric plexus neurons of the small intestine and colon (Mao et al. 1996; Cox et al. 2001; Wang et al. 2010). They delay gastric emptying and are mediators of the ileal break, also inhibit gastric and pancreatic secretion and stimulate the absorption of water and electrolytes. In some ways, they provide an integrated functional defence against luminal harmful factors including toxins.

Some diarrhetic agents have been proven to alter NPY and PYY expression (Moriya et al. 2010). Previous studies (Valdiglesias et al. 2012; Louzao et al. 2015) have also shown an impairment of NPY production on SH-SY5Y neuroblastoma cell line when treated with OA. In agreement with these results, in vivo, we found a decrease in small intestine NPY concentration mainly after 24 h of oral OA administration. This could be related to the increase in intestinal fluid secretion observed in necropsy. Other diarrhoeic compounds, for example, Cholera toxin, induce hyperexcitability of secretomotor neurons in enteric pathways (Gwynne et al. 2009), while intraarterial infusion of the neuropeptide notably reduced this enterotoxin-evoked fluid secretion in cats (Sjoqvist et al. 1988). However, pre-treatment with NPY did not reduce OA-induced diarrhoea and intestinal fluid accumulation was only modestly improved. This was further supported by similar results obtained at a shorter time of exposure and with lower OA dose.

PYY exerts multiple physiological effects on the gastrointestinal tract (El-Salhy et al. 2020). PYY inhibited Prostaglandin E_2_ (PGE_2_) and Vasoactive Intestinal Peptide that stimulated intestinal water secretion in the human small intestine being a defence against diarrhoea (Moriya et al. 2010; Roze et al. 1997). We detected a reduction in PYY in the small intestine of mice 24 h after receiving OA but this decrease was not clear after 6 h of treatment. Besides, Y_2_ agonist PYY(3–36) administration had no effect on OA-induced diarrhoea although it was previously reported that PYY prevented faecal pellet output caused by dimethyl-PGE_2_ (Moriya et al. 2010) or inhibited propulsive colonic motor function through Y_2_ receptor in conscious mice (Wang et al. 2010).

Therefore, the addition of Y receptor agonist NPY (Y_1_ and Y_2_ receptors) or PYY(3–36) (Y_2_ receptor) induced almost no improvement on intestinal and stomach fluid accumulation even in mice that had no faeces. Besides, the lack of a robust delay or prevention of OA-induced diarrhoea by targeting pro-absorptive peptides suggests that other enteric nervous pathways should be involved.

Serotonin is an endogenous signalling molecule involved in the regulation of fluid and mucus secretion as well as regulation of ion transport in gastrointestinal tract (Banskota et al. 2019) capable of altering intestinal motility and implicated in diarrhoea outcome (Thiagarajah et al. 2015; Camilleri et al. 2017; Hu and Spencer 2018). In fact, several diarrhoeagenic agents have been strongly related to this molecule (Ha et al. 2021; Westerberg et al. 2018; Singhal et al. 2017). Serotonin effects are achieved through the action on epithelial 5-HT_2_ receptor and neuronal 5-HT_1_, 5-HT_3_ and 5-HT_4_ receptors (Fidalgo et al. 2013). Cyproheptadine (CPH) a 5-HT_1_ and 5-HT_2_ receptor antagonist/inverse agonist has potent antiserotoninergic effects decreasing contraction of longitudinal smooth muscles of small intestine in mice (Fida et al. 2000). Our experiments are the first to evaluate the effects of 5-HT receptor antagonist during OA intoxication in vivo. In our CPH dose–response study at 2 h, the highest dose (10 mg/kg CPH) prevented the phycotoxin effects regarding diarrhoea, even lower doses (0.1 and 1 mg/kg CPH) delayed OA-induced diarrhoea onset. Average of OA-triggered diarrhoea outbreak was 33 ± 2.3 min. At this time, serotonin measured in large intestine was slightly elevated in OA-treated mice and remains high in CPH pre-treated mice. Interestingly, our results showed no diarrhoea in mice pre-treated with CPH at any dose; meanwhile, OA-treated mice have this symptom 30 min after receiving the toxin. Other potent diarrhoeagenic compounds, such as Cholera toxin, cause the symptom by increasing the secretion of water into intestinal lumen. It can modify gastrointestinal motility by stimulating secretomotor neurons leading to the release of serotonin from enteroendocrine cells (Spencer and Hu 2020). This enterotoxin has been reported to prompt hypersecretion via 5-HT release in human (Bearcroft et al. 1996) and rat jejunum (Beubler et al. 1989) as well as in vitro primary enterochromaffin tumour cells (Hagbom et al. 2011). In accordance, 5-HT_2_ and 5-HT_3_ receptors have been described to mediate this toxin-induced fluid secretion in rat jejunum (Beubler et al. 1989; Beubler and Horina 1990) while the antagonist of 5-HT_2_ receptor ketanserin ameliorated fluid secretion evoked by the compound in rats (Harville and Dreyfus 1995). Our results showed that an increase in fluid secretion occurs within 30 min exposure of OA. This early secretion can be partially inhibited by CPH, making the contents of the large intestine normal (this was not achieved with pre-treatments with NPY or PYY), suggesting a role for serotonin as a mediator during this stage. In relation to this, it was reported that CPH has a direct effect on the inhibition of electrogenic ion secretion in the intestinal epithelium (Meddah et al. 2014). This effect could also explain the clear improvement of clinical signs and major gross findings of dilation of the large bowel appreciated during necropsy in CPH pre-treated mice. Therefore, CPH inhibited the OA-induced diarrhoea by blocking serotonin activity on 5-HT receptors. All these findings entail an indication of neuronal signalling mediation in the pathophysiology of DSP in mice, mainly involving 5-HT activity.

## Conclusions

The fast symptoms OA causes during shellfish poisoning in humans (diarrhoea, nausea, vomiting and abdominal pain) suggested a neurogenic component. We determined that diarrhoea onset is an all-or-none response independent from the given OA dose. Moreover, we showed the inhibitory effect of cyproheptadine on OA-induced diarrhoea, involving serotonin in the toxicity mechanism. This work evidences OA effect mainly on serotonin action and leads to gain further insight into the mechanism triggering diarrhoea. Also, it opens the possibility to further research the OA effect in the enteric nervous system and the enteroendocrine cross-talk.

## Supplementary Information

Below is the link to the electronic supplementary material.

Supplementary file1 (DOCX 4643 KB)

## Data Availability

The datasets generated during and/or analysed during the current study are available from the corresponding author on reasonable request.

## Code availability

Graphpad Prism and RStudio were the software used to perform statistical analysis.
