# Supplementary material for: Serotonin involvement in okadaic acid-induced diarrhoea in vivo
Source: Arch Toxicol. 2021 Jun 20;95(8):2797–813. doi: 10.1007/s00204-021-03095-z (PMC8298366; doi:10.1007/s00204-021-03095-z)
Supplement: Supplementary file 1 — Supplementary file1 (DOCX 4643 KB) [file 204_2021_3095_MOESM1_ESM.docx]

**Serotonin involvement in okadaic induced diarrhoea *in vivo***

M. Carmen Louzao^1,^*, Celia Costas^1^, Paula Abal^1^, Toshiyuki Suzuki^2^, Ryuichi Watanabe^2^, Natalia Vilariño^1^, Cristina Carrera^1^, Andrea Boente-Juncal^1^, Carmen Vale^1^, Mercedes R. Vieytes^3^, Luis M. Botana^1^.

^1^ Departamento de Farmacología, Facultad de Veterinaria, Universidad de Santiago de Compostela, Lugo 27002, Spain

^2^ Fisheries Technology Institute, National Research and Development Agency, Japan Fisheries Research and Education Agency, Yokohama 236-8648, Japan.

^3^ Departamento de Fisiología, Facultad de Veterinaria, Universidad de Santiago de Compostela, Lugo 27002, Spain

* Correspondence: [mcarmen.louzao@usc.es](mailto:mcarmen.louzao@usc.es) (M.C. Louzao)

**a**

**b**


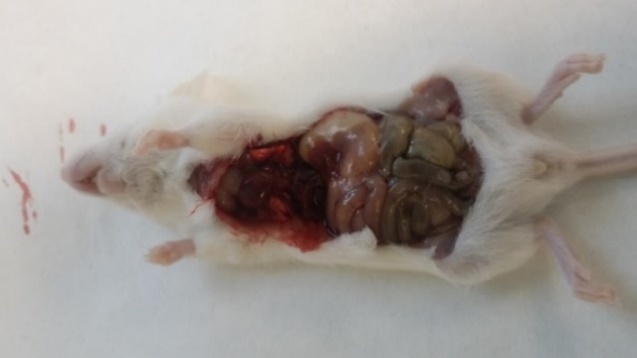

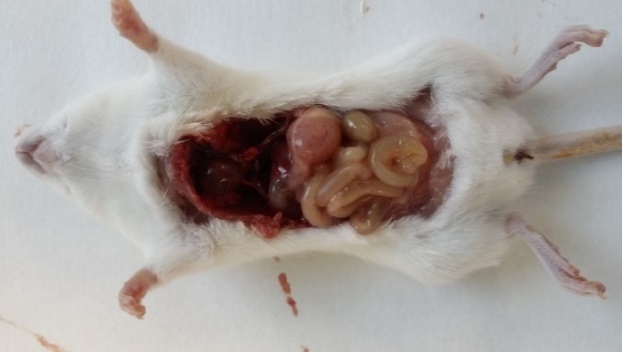

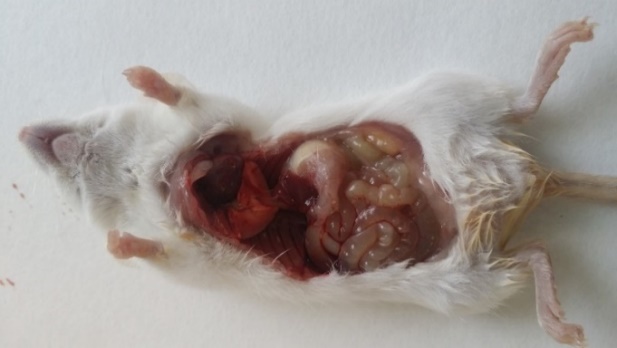

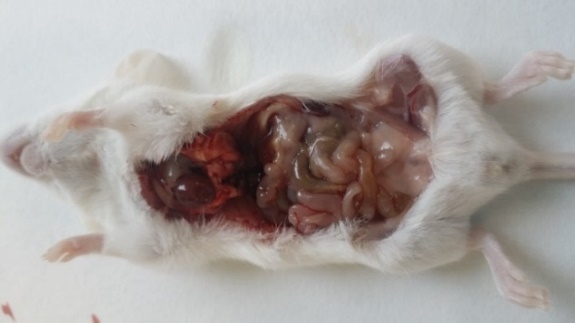


**Control**

**OA**

**NPY**

**NPY-OA**

**d**

**c**

**Fig. S1** NPY effect on OA poisoning at 6 h of treatment. Animals received 107 µg/kg NPY 15 min prior to 550 µg/kg OA administration. (**a**) Body weight variation, (**b**) food intake and (**c**) water intake during the assessment. (**d**) Representative images of the abdominal cavity. Graphs (**a**, **b** and **c**) show mean ± SEM (n=4 performed in duplicate). ANOVA followed by Bonferroni Multiple Comparison Test were executed. Statistical significances are indicated by asterisks set over the line between treatments, so that *P<0.05 is indicated in (**b**). In (**c**), different sign indicates differences between groups of *P<0.05

**a**

**b**

**d**


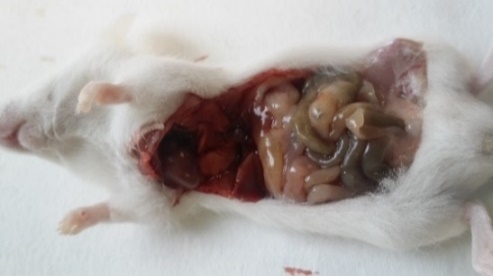

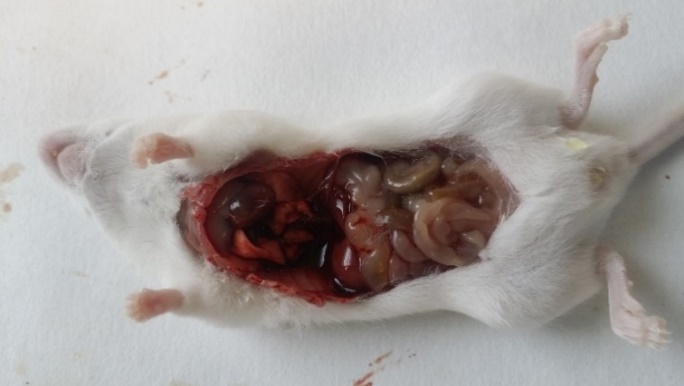

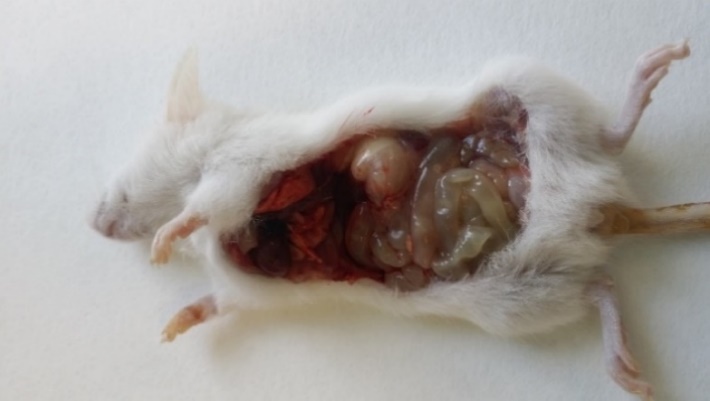

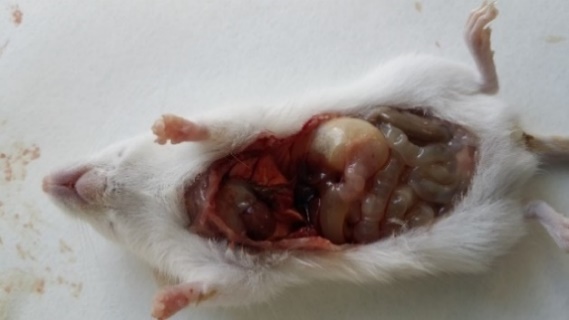


**Control**

**OA**

**PYY(3-36)**

**PYY(3-36)-OA**

**c**

**Fig. S2** PYY(3-36) pre-treatment effect on OA at 6 h. *In vivo* assay testing 1 mg/kg PYY(3-36) and 550 µg/kg OA on mice. (**a**) Body weight variation, (**b**) food intake and (**c**) water intake along the experiment. (**d**) Macroscopic examination representative photos of treatments. Mean ± SEM (n=3 performed in duplicate) is presented. No statistical significance was detected by one-way ANOVA

**a**

**b**

**Control**


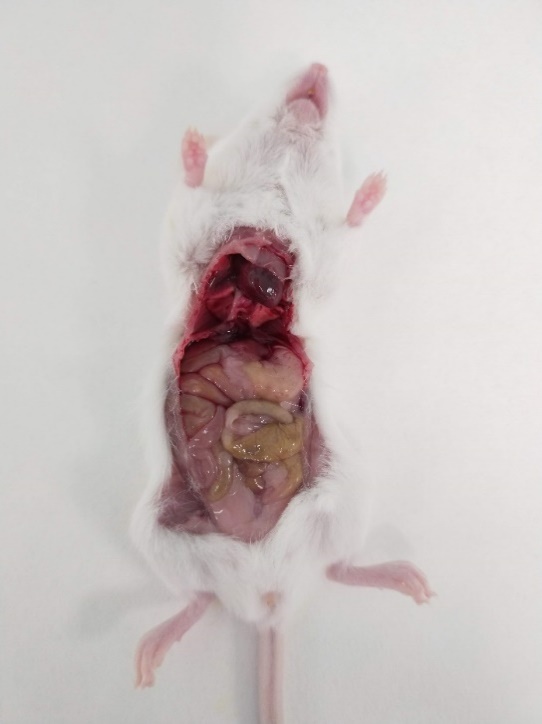


**NPY**


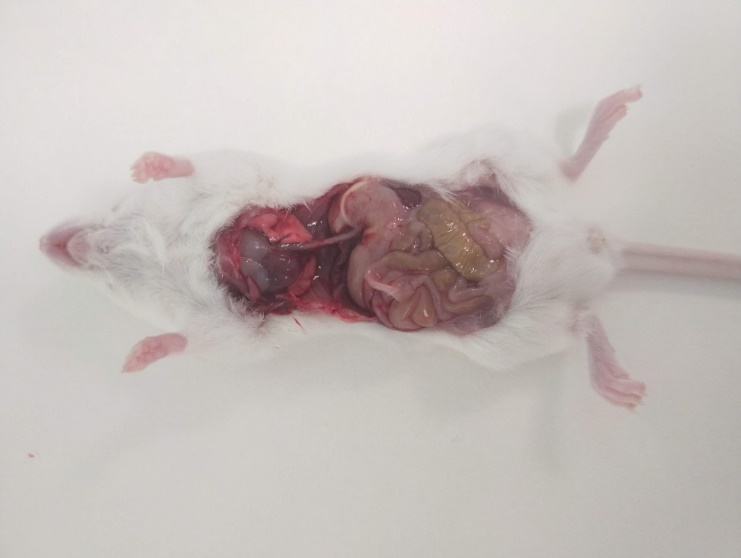


**PYY(3-36)**


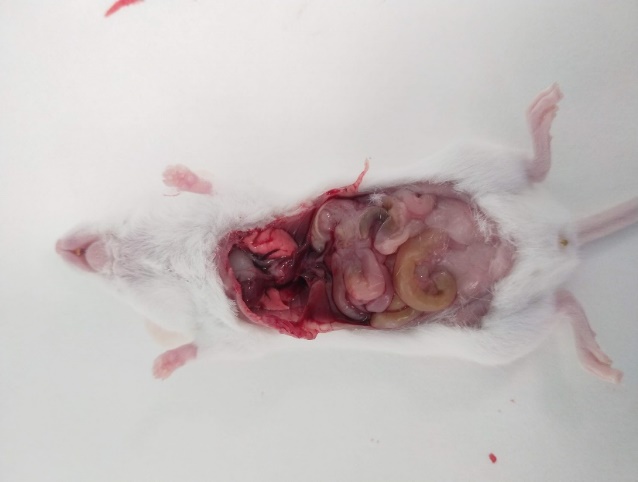


**OA**

**NPY-OA**

**PYY(3-36)-OA**


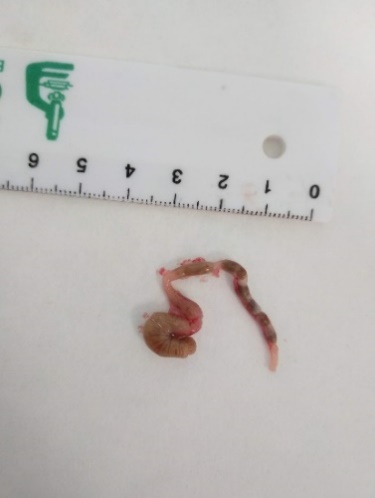

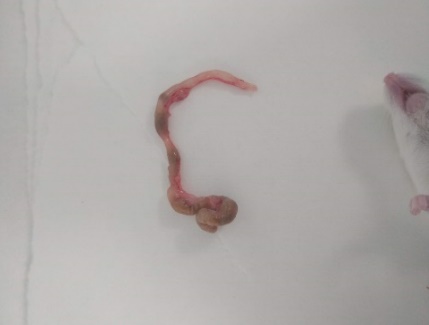

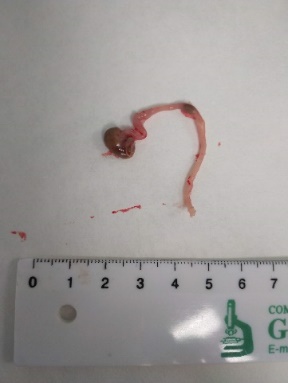

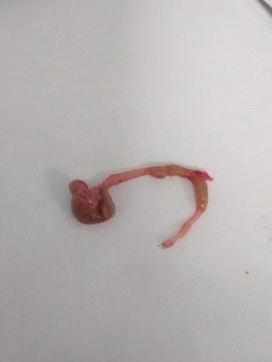

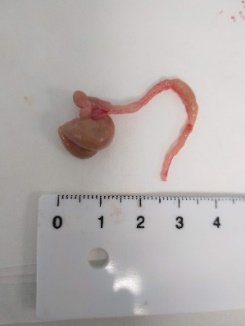

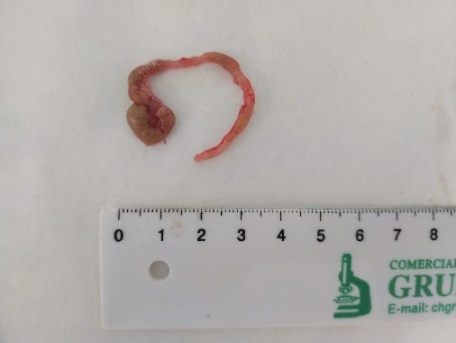

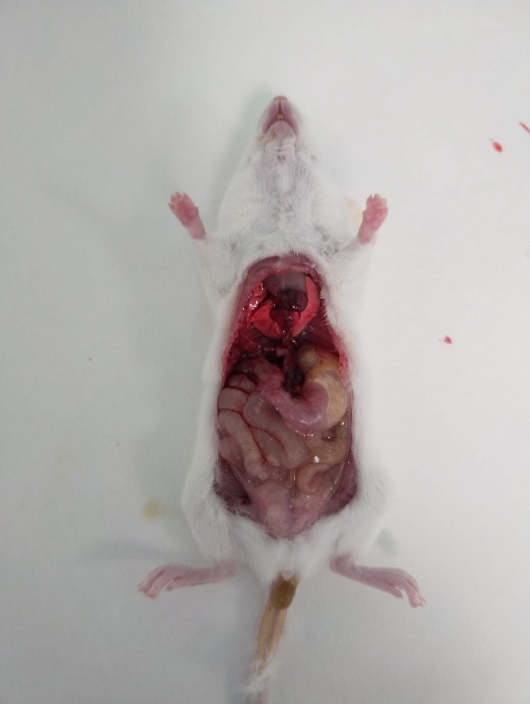

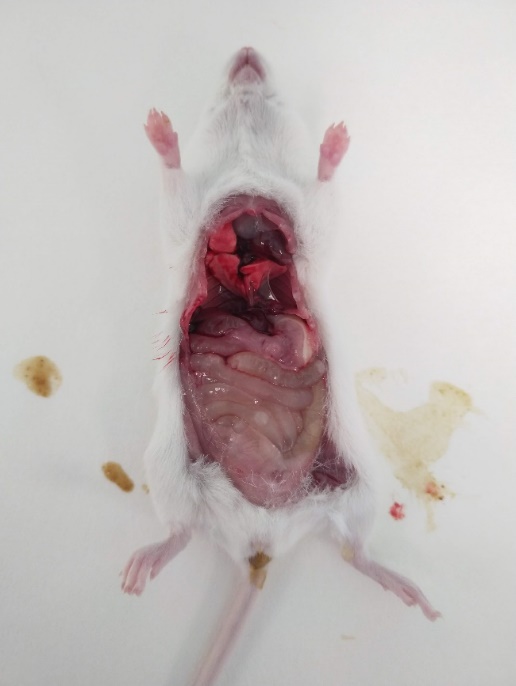

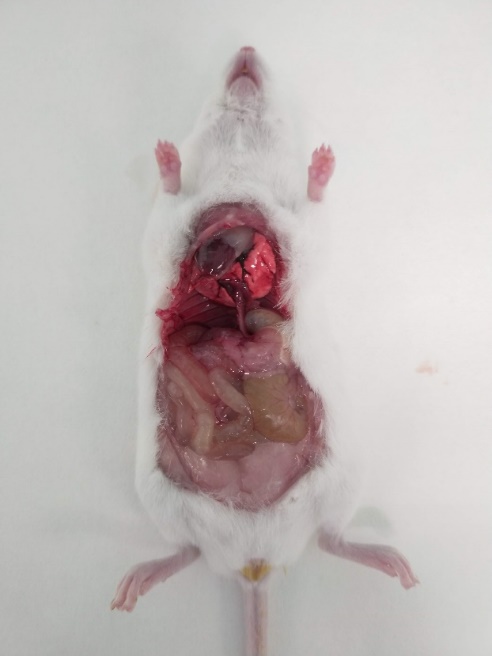


**c**

**Fig. S3** NPY or PYY(3-36) pre-treatment effects on OA after 2 h of toxin administration. NPY (107 µg/kg) or PYY(3-36) (1 mg/kg) were injected 15 min prior to OA (250 µg/kg) oral treatment. (**a**) Body weight variance and (**b**) food intake during the experiment were measured. (**c**) Representative images of the abdominal cavity and the large intestine at the end of the treatment. Data are presented as mean ± SEM (n=3 of duplicates). Kruskal-Wallis analysis resulted in no significant differences between groups

**a**

**b**


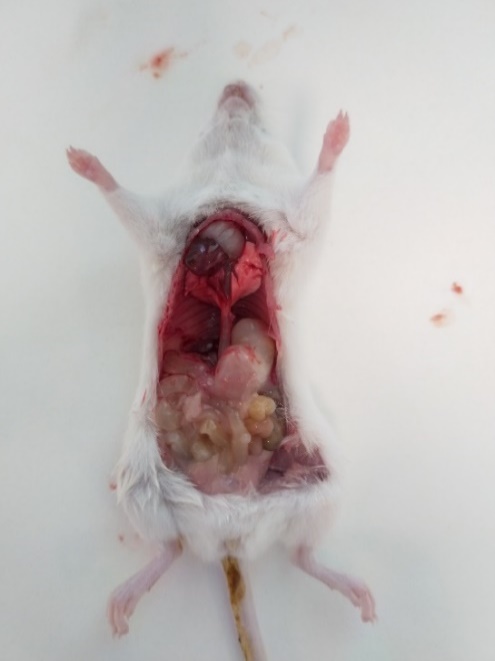


**d**


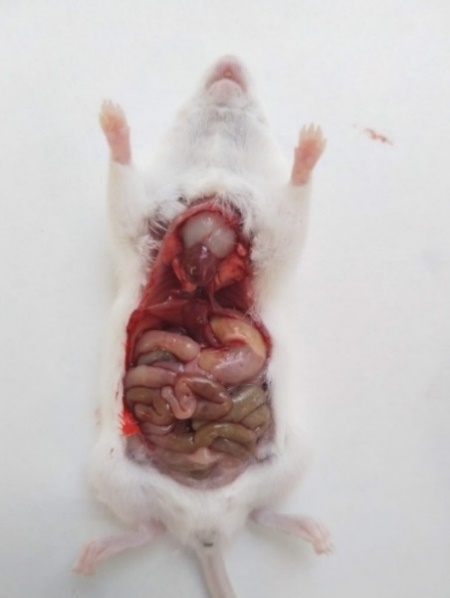

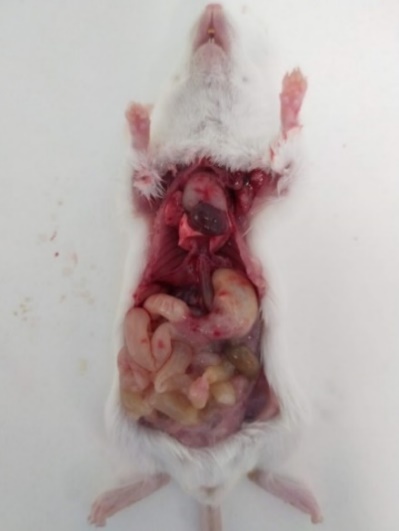

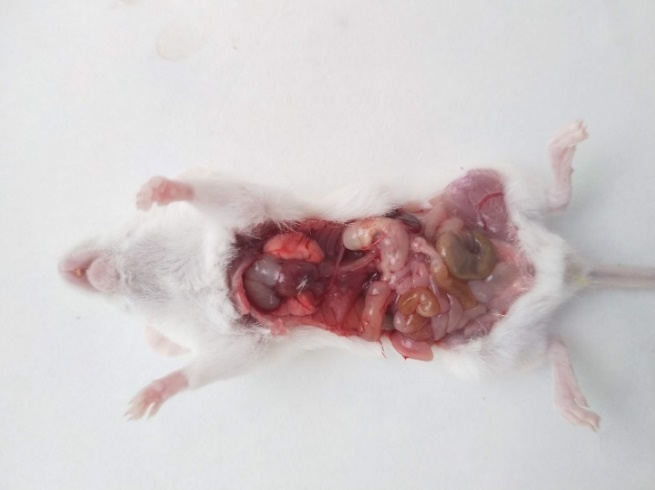

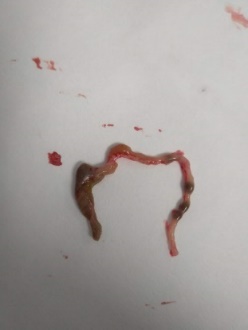

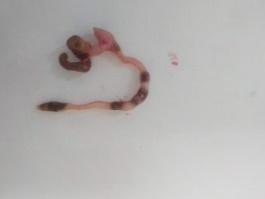

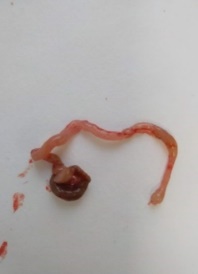

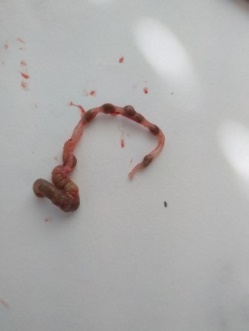


**Control**

**OA**

**CPH**

**CPH-OA**

**c**

**Fig. S4** CPH effect on OA intoxication after 6 h of treatment. Animals were treated with 3 mg/kg CPH 30 min before 250 µg/kg OA administration for 6 h. (**a**) Body weight balance, (**b**) food intake and (**c**) water intake during the assessment were measured. (**d**) Representative images of abdominal cavity and large intestine at the end of the experiment. Mean ± SEM (n=7 of duplicates) is presented. Data were analysed by ANOVA and Bonferroni Multiple Comparison Test. Significant differences are indicated by asterisks centred over the line between treatments as *P<0.05


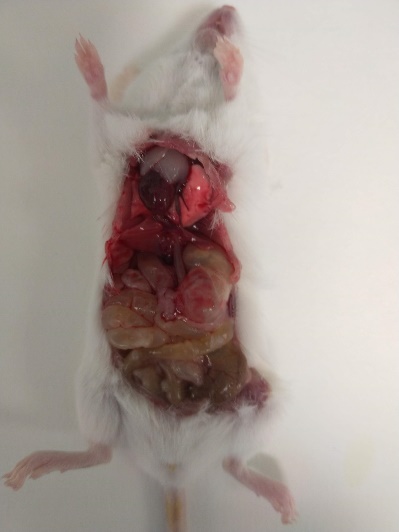

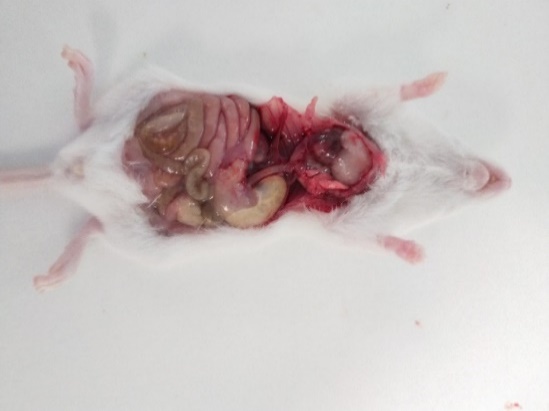

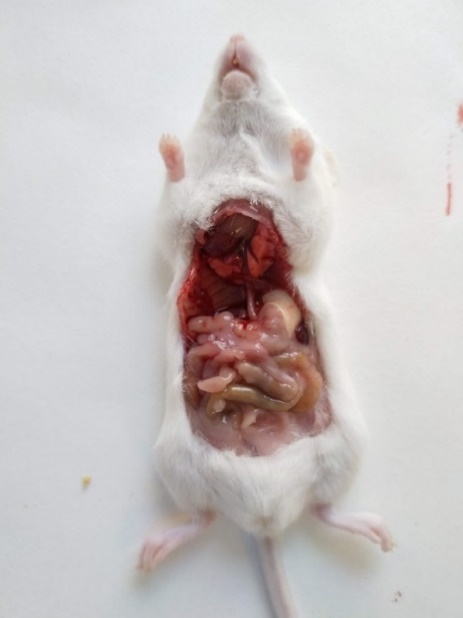

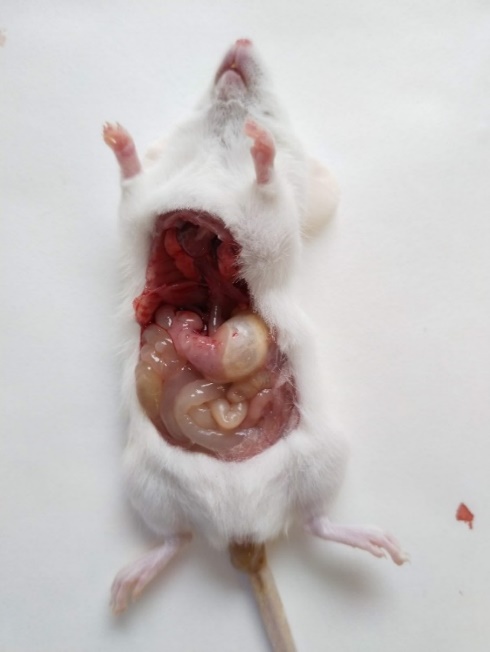

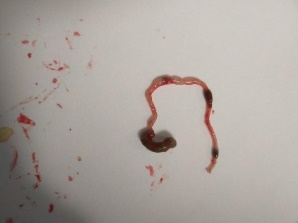

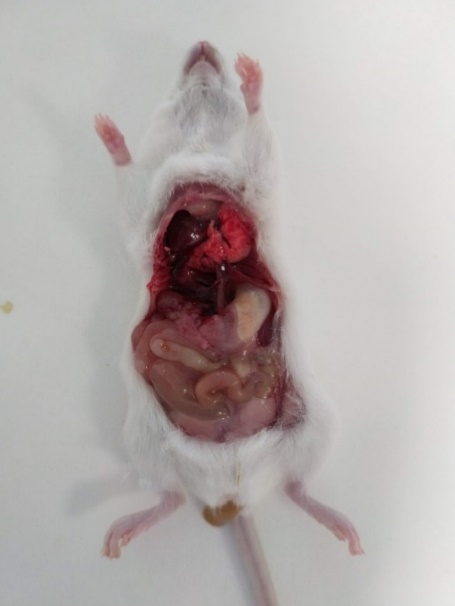

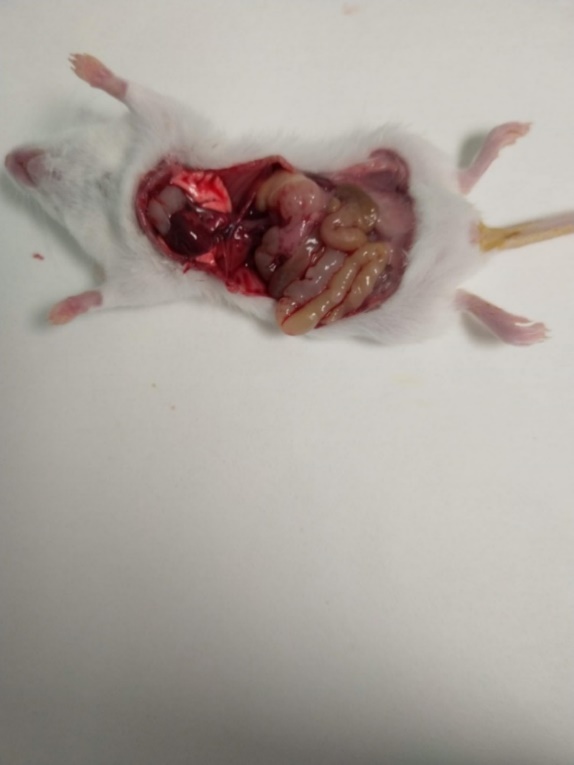

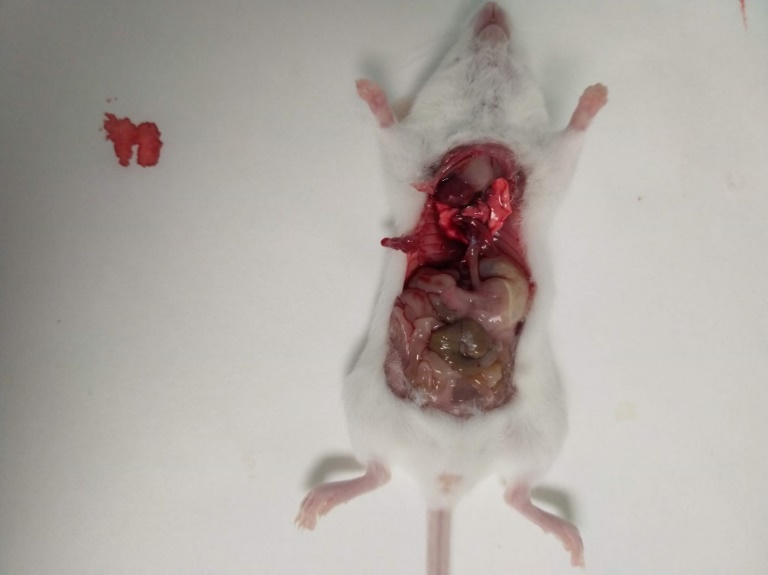

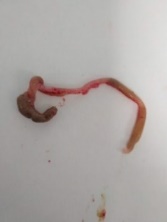

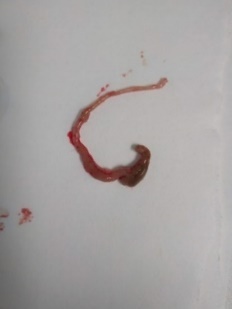

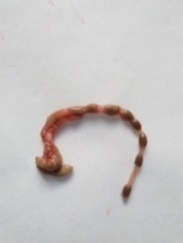

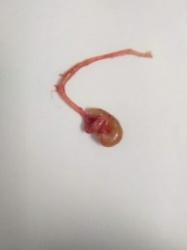

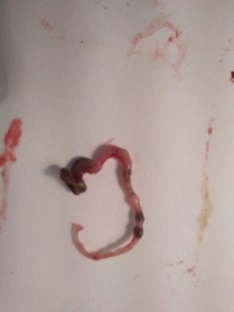

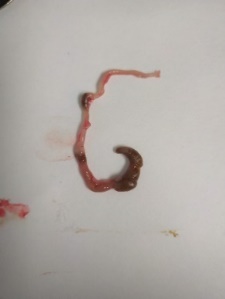


**Control**

**OA**

**CPH**

**0.1 mg/kg CPH**

**OA**

**10 mg/kg CPH**

**OA**

**1 mg/kg CPH**

**OA**

**3 mg/kg CPH**

**OA**

**c**

**a**

**b**

**Fig. S5** Dose-response of CPH assessment on OA treatment for 2 h. Mice received 0.1, 1, 3 or 10 mg/kg CPH and 30 min later 250 µg/kg OA. Effects of treatments on (**a**) body weight and (**b**) food intake along the experiment. Values are expressed as mean ± SEM (n=3 of duplicates). (**c**) Representative images of mice stomach and small intestine as well as large intestine at the end of the experiment. One-way ANOVA (**a**) or Kruskal-Wallis (**b**) were conducted, significant differences between groups are stated as *P<0.05

**a**

**b**

**Fig. S6** CPH dose-response effect on OA-induced diarrhoea at the time it occurred. Mice were pre-treated with 0.1, 1 or 6 mg/kg CPH and 30 min later with 250 µg/kg OA. The end of the experiment was set at the time OA triggered diarrhoea. Effects of treatments on (**a**) body weight modification and (**b**) food intake in mice. Mean ± SEM (n=3 of duplicates) are presented. ANOVA followed by Bonferroni Multiple Comparison, resulting in no significant differences

**Table S1** Effect of NPY pre-treatment on symptomatology of OA-treated animals for 6 h (%)

| Symptoms | Control | NPY  107 µg/kg | OA  550 µg/kg | 107 µg/kg NPY  550 µg/kg OA |
| --- | --- | --- | --- | --- |
| Apathy | 0 | 0 | 40 | 54.5 |
| Piloerection | 0 | 25 | 80 | 63.6 |
| Cyanosis | 0 | 25 | 100 | 63.6 |
| Spasms | 0 | 0 | 40 | 27.3 |
| On hind legs | 0 | 0 | 60 | 9.1 |
| Squint eyes | 0 | 25 | 80 | 81.8 |
| Dyspnea | 0 | 0 | 0 | 9.1 |
| Diarrhoea | 0 | 0 | 100 | 100 |
| Mortality | 0 | 0 | 20 | 9.1 |

**Table S2** Effect of PYY pre-treatment on clinical signs of mice treated with OA for 6 h (%)

| Symptoms | Control | PYY(3-36)  1 mg/kg | OA  550 µg/kg | 1 mg/kg PYY(3-36)  550 µg/kg OA |
| --- | --- | --- | --- | --- |
| Apathy | 0 | 0 | 66.7 | 71.4 |
| Piloerection | 0 | 0 | 100 | 71.4 |
| Cyanosis | 0 | 0 | 33.3 | 57.1 |
| Spasms | 0 | 0 | 33.3 | 0 |
| On-hind legs | 0 | 0 | 100 | 0 |
| Squint eyes | 0 | 0 | 100 | 85.7 |
| Dyspnea | 0 | 0 | 0 | 14.3 |
| Diarrhoea | 0 | 0 | 100 | 85.7 |
| Mortality | 0 | 0 | 0 | 14.3 |

**Table S3** NPY or PYY(3-36) effect on the symptomatology induced by OA at 2 h of treatment (%)

| **Symptoms** | **Control** | **NPY**  **107 µg/kg** | **PYY(3-36)**  **1 mg/kg** | **OA**  **250 µg/kg** | **107 µg/kg NPY**  **+ 250 µg/kg OA** | **1 mg/kg PYY(3-36)**  **+ 250 µg/kg OA** |
| --- | --- | --- | --- | --- | --- | --- |
| Apathy | 0 | 0 | 0 | 66.6 | 100 | 83.3 |
| Piloerection | 0 | 0 | 0 | 100 | 83.3 | 50 |
| Cyanosis | 0 | 0 | 0 | 0 | 0 | 16.7 |
| Spasms | 0 | 0 | 0 | 0 | 0 | 16.7 |
| On-hind legs | 0 | 0 | 0 | 33.3 | 50 | 33.3 |
| Squint eyes | 0 | 0 | 0 | 100 | 100 | 83.3 |
| Dyspnea | 0 | 0 | 0 | 0 | 0 | 0 |
| Hunched position | 0 | 0 | 0 | 33.3 | 33.3 | 33.3 |
| Diarrhoea | 0 | 0 | 0 | 100 | 83.3 | 83.3 |
| Mortality | 0 | 0 | 0 | 0 | 0 | 0 |

**Table S4** Effect of CPH pre-treatment on mice symptomatology after treatment with OA for 6 h (%)

| Symptoms | Control | CPH  3 mg/kg | OA  250 µg/kg | 3 mg/kg CPH  250 µg/kg OA |
| --- | --- | --- | --- | --- |
| Apathy | 0 | 0 | 71.4 | 92.3 |
| Piloerection | 0 | 0 | 28.6 | 46.1 |
| Cyanosis | 0 | 0 | 14.3 | 7.7 |
| Spasms | 0 | 0 | 14.3 | 7.7 |
| On-hind legs | 0 | 0 | 57.1 | 15.4 |
| Squint eyes | 0 | 0 | 57.1 | 76.9 |
| Dyspnea | 0 | 0 | 0 | 15.4 |
| Hunched position | 0 | 0 | 57.1 | 30.8 |
| Diarrhoea | 0 | 0 | 100 | 61.5 |
| Mortality | 0 | 0 | 0 | 0 |

**Table S5** Effect of pre-treatment with different doses of CPH on symptomatology monitored in OA treated mice until 2 h (%)

| Symptoms | Control | CPH  (mg/kg) | OA  250 µg/kg | CPH (mg/kg) + 250 µg/kg OA | | | |
| --- | --- | --- | --- | --- | --- | --- | --- |
|  |  |  |  | **0.1** | **1** | **3** | **10** |
| Apathy | 0 | 0 | 37.5 | 33.3 | 66.7 | 14.3 | 66.7 |
| Piloerection | 33.3 | 0 | 37.5 | 100 | 100 | 0 | 33.3 |
| Cyanosis | 0 | 0 | 25 | 33.3 | 66.7 | 0 | 33.3 |
| Spasms | 0 | 0 | 50 | 0 | 0 | 28.6 | 0 |
| On-hind legs | 0 | 0 | 50 | 33.3 | 0 | 0 | 0 |
| Squint eyes | 16.7 | 0 | 25 | 33.3 | 33.3 | 0 | 100 |
| Dyspnea | 0 | 0 | 0 | 0 | 0 | 0 | 0 |
| Hunched position | 0 | 0 | 50 | 66.7 | 66.7 | 0 | 33.3 |
| Diarrhoea | 0 | 0 | 87.5 | 66.7 | 100 | 0 | 0 |
| Mortality | 0 | 0 | 0 | 0 | 0 | 0 | 0 |

**Table S6** Effect of pre-treatment with different doses of CPH on clinical signs exhibited by short exposure to OA (%)

| **Symptoms** | **Control** | **CPH**  **(mg/kg)** | **OA**  **250 µg/kg** | **CPH (mg/kg) + 250 µg/kg OA** | | |
| --- | --- | --- | --- | --- | --- | --- |
|  |  |  |  | **0.1** | **1** | **6** |
| Apathy | 0 | 0 | 75 | 33.3 | 33.3 | 33.3 |
| Piloerection | 0 | 0 | 50 | 33.3 | 0 | 0 |
| Cyanosis | 0 | 0 | 0 | 0 | 0 | 0 |
| Spasms | 0 | 0 | 0 | 33.3 | 0 | 0 |
| On-hind legs | 0 | 0 | 0 | 0 | 0 | 0 |
| Squint eyes | 0 | 0 | 50 | 33.3 | 0 | 33.3 |
| Dyspnea | 0 | 0 | 25 | 0 | 0 | 0 |
| Hunched position | 0 | 0 | 0 | 0 | 0 | 0 |
| Diarrhoea | 0 | 0 | 75 | 0 | 0 | 0 |
| Mortality | 0 | 0 | 0 | 0 | 0 | 0 |
